# Alzheimer risk gene product Pyk2 suppresses tau phosphorylation and phenotypic effects of tauopathy

**DOI:** 10.1186/s13024-022-00526-y

**Published:** 2022-05-03

**Authors:** A. Harrison Brody, Sarah Helena Nies, Fulin Guan, Levi M. Smith, Bandhan Mukherjee, Santiago A. Salazar, Suho Lee, Tu Kiet T. Lam, Stephen M. Strittmatter

**Affiliations:** 1 Cellular Neuroscience, Neurodegeneration and Repair Program, Departments of Neurology and Neuroscience, Yale School of Medicine, New Haven, CT USA; 2 Graduate School of Cellular and Molecular Neuroscience, University of Tübingen, D-72074 Tübingen, Germany; 3 Department of Molecular Biophysics and Biochemistry, Yale School of Medicine, New Haven, CT USA; 4 Keck MS & Proteomics Resource, Yale School of Medicine, New Haven, CT USA

**Keywords:** Alzheimer’s disease, Fronto-temporal dementia, Tauopathy, *PTK2B*, Pyk2, C1q

## Abstract

**Background:**

Genetic variation at the *PTK2B* locus encoding the protein Pyk2 influences Alzheimer’s disease risk. Neurons express Pyk2 and the protein is required for Amyloid-β (Aβ) peptide driven deficits of synaptic function and memory in mouse models, but Pyk2 deletion has minimal effect on neuro-inflammation. Previous in vitro data suggested that Pyk2 activity might enhance GSK3β-dependent Tau phosphorylation and be required for tauopathy. Here, we examine the influence of Pyk2 on Tau phosphorylation and associated pathology.

**Methods:**

The effect of Pyk2 on Tau phosphorylation was examined in cultured Hek cells through protein over-expression and in iPSC-derived human neurons through pharmacological Pyk2 inhibition. PS19 mice overexpressing the P301S mutant of human Tau were employed as an in vivo model of tauopathy. Phenotypes of PS19 mice with a targeted deletion of Pyk2 expression were compared with PS19 mice with intact Pyk2 expression. Phenotypes examined included Tau phosphorylation, Tau accumulation, synapse loss, gliosis, proteomic profiling and behavior.

**Results:**

Over-expression experiments from Hek293T cells indicated that Pyk2 contributed to Tau phosphorylation, while iPSC-derived human neuronal cultures with endogenous protein levels supported the opposite conclusion. In vivo, multiple phenotypes of PS19 were exacerbated by Pyk2 deletion. In Pyk2-null PS19 mice, Tau phosphorylation and accumulation increased, mouse survival decreased, spatial memory was impaired and hippocampal C1q deposition increased relative to PS19 littermate controls. Proteomic profiles of Pyk2-null mouse brain revealed that several protein kinases known to interact with Tau are regulated by Pyk2. Endogenous Pyk2 suppresses LKB1 and p38 MAPK activity, validating one potential pathway contributing to increased Tau pathology.

**Conclusions:**

The absence of Pyk2 results in greater mutant Tau-dependent phenotypes in PS19 mice, in part via increased LKB1 and MAPK activity. These data suggest that in AD, while Pyk2 activity mediates Aβ-driven deficits, Pyk2 suppresses Tau-related phenotypes.

**Supplementary Information:**

The online version contains supplementary material available at 10.1186/s13024-022-00526-y.

## Background

Alzheimer’s disease (AD), the most common cause of dementia, is the 6th leading cause of death overall and the 5th leading cause of death in individuals 65 years and older in the United States [1]. Currently, there are an estimated 6.2 million individuals living with AD in the US, a number that is expected to increase more than two-fold by 2050 [1]. With no therapeutic interventions known to slow or halt AD progression, AD is situated to overwhelm existing global health care infrastructure.

The tyrosine kinase Pyk2 (*PTK2B*) has been identified in multiple genome wide association studies (GWAS) as a risk factor for late-onset AD (LOAD) [2–7], and at least one AD-associated *PTK2B* variant (rs28834970) results in increased Pyk2 expression in human peripheral blood monocytes [8]. Biochemically, Pyk2 demonstrates increased activity in both wild-type mouse brain slices treated with oligomeric amyloid beta (Aβo) and in brain lysates from aged APPswe/PS1ΔE9 (APP/PS1) transgenic mice [9, 10].

Recently, our group reported that genetic deletion of Pyk2 rescues a number of Aβ-associated phenotypes in APP/PS1 animals including memory impairment, synapse loss, astrogliosis and impaired synaptic plasticity [11]. Mechanistically, Aβo-induced dendritic spine loss in mouse primary hippocampal neurons was dependent on the expression of Pyk2 [12]. In the presence of Aβo, the Aβo receptor PrP^C^ increases its association with the Aβo co-receptor mGluR5, which triggers the intracellular release of Pyk2 from the PrP^C^-mGluR5 signaling complex [9, 13, 14]. Once dissociated from mGluR5, activated Pyk2 participates in aberrant, downstream Aβo-induced signaling events, including the activation of RhoA and JNK, contributing to spine loss and apoptosis, respectively [12, 15–20].

While Pyk2’s contribution to pathological Aβ signaling is relatively well-described, Pyk2’s role in regulating Tau is less defined, despite strong correlative evidence for such a role. GSK3β, for example, a kinase known to phosphorylate Tau at a number of pathophysiologically-relevant residues [21–26], is activated by Pyk2 [27–29]. Pyk2 has also been shown to interact biochemically with Tau in Hek293 cells and to colocalize with hyperphosphorylated Tau fibrils in both AD brains and Tau transgenic mice [30]. Additional evidence suggests that Pyk2 may phosphorylate Tau directly. Pyk2 co-localizes with Tau in mouse primary hippocampal neurons, phosphorylates Tau at Y18 in vitro and augments the phosphorylation of Tau at Y18 when over-expressed in MAPT P301L transgenic mice [31].

Notwithstanding an abundance of correlative data suggesting a role for Pyk2 in regulating Tau, existing evidence of Pyk2’s ability to phosphorylate Tau either directly or indirectly has relied exclusively on Pyk2 over-expression. To the best of our knowledge, the results reported here represent the first attempt at elucidating Pyk2’s ability to regulate Tau phosphorylation and Tau pathology through the modulation of endogenous Pyk2. While our results confirm that Pyk2 over-expression contributes to Tau phosphorylation, suppression of endogenous Pyk2 activity through either pharmacological inhibition or genetic deletion *increases* the phosphorylation of Tau at a number of pathophysiologically-relevant residues. When crossed with Pyk2^−/−^ animals, hemizygous PS19 (MAPT P301S, 1N4R) transgenic mice demonstrate augmented Tau pathology, decreased survival, impaired spatial memory and increased hippocampal C1q deposition. Phospho-proteomic analysis of hippocampal synaptosomes reveals a number of putative Pyk2-modulated regulators of Tau, one of which (LKB1) is further validated here. LKB1 positively regulates the activity of the Tau kinase p38 MAPK [32–38], and the activities of both kinases are suppressed by endogenous levels of Pyk2. Together, these results suggest a protective role for Pyk2 against Tau phosphorylation, Tau pathology and Tau-induced behavioral deficits, in part through the suppression of LKB1 and p38 MAPK activity.

## Methods

### Plasmid DNA constructs

Tau and Pyk2 sequences were subcloned into an AAV-CAG-GFP vector (RRID:Addgene_28014) and GSK3β and Fyn sequences subcloned into a pcDNA3.0 vector which served as a negative transfection control.

### Hek293T cell culture and transfection

Human embryonic kidney 293T (Hek293T, RRID:CVCL_QW54) cells were cultured in DMEM (Gibco #11965092) with 10% FBS (Gibco #16000044) and incubated at a constant 37 °C with 5% CO_2_. For protein over-expression, Hek293T cells were transfected with appropriate DNA constructs (0.5 μg DNA/well in a 12-well plate) using Lipofectamine 3000 reagent (Invitrogen #L3000001). Cells were harvested in 1% Triton X-100 containing 50 mM Tris, 150 mM NaCl and 1 mM EDTA with protease (Roche #11836170001) and phosphatase (Roche #4906845001) inhibitors. Lysates were spun at 14,000×*g* for 10 min at 4 °C and Triton X-100-soluble supernatants were boiled in Laemmli sample buffer (Bio-Rad #1610747) at 95 °C for 10 min.

### Animals

B6;C3-Tg (Prnp-MAPT*P301S) PS19Vle/J (RRID:IMSR_JAX:008169) mice purchased from Jackson Laboratories (JAX) were backcrossed over 5 generations to a C57BL/6 J background to obtain hemizygous PS19^0/+^ animals. Pyk2^−/−^ mice, backcrossed to a C57BL/6J background over 10 generations by Schlessinger and colleagues [39] (RRID:MGI_3584536), were generously provided by Dr. David Schlaepfer (University of California–San Diego). Hemizygous PS19^0/+^ and Pyk2^+/−^ mice were paired to obtain WT, Pyk2^−/−^, PS19^0/+^ and Pyk2^−/−^; PS19^0/+^ mice. All experiments used littermate control mice with no preference for either female or male animals. Comparisons of male and female outcomes by group were conducted post hoc. All protocols including animal husbandry, genotyping, behavioral testing and euthanasia were approved by the Yale University Institutional Animal Care and Use Committee (IACUC). Animals were housed in groups of 2–5 mice per cage with access to food and water ad libitum. 12-h light/dark cycles were maintained throughout the duration of animal housing with light periods beginning consistently at 7 am.

### Acute brain slice pharmacology

4.5–5.5-month-old PS19^0/+^ mice were sacrificed via live decapitation in accordance with Yale University’s Institutional Animal Care and Use Committee standards. Brains were dissected on ice and sectioned using a Leica WT1000S Vibratome in ice-cold, oxygenated (95% O_2_, 5% CO_2_) artificial cerebrospinal fluid (aCSF) containing 119 mM NaCl, 2.5 mM KCl, 2.7 mM MgSO_4_, 26 mM NaHCO_3_, 11 mM D-glucose and 1.25 mM NaH_2_PO_4_. Three 400-μm-thick coronal sections (containing the three most rostral sections of hippocampus) were collected per brain. The two hemispheres of each section were divided medially and slices enriched for hippocampal tissue by removal of the ventral half of each section using a sharp razor blade. Hippocampal-enriched brain slices were transferred to a radial-arm incubation chamber (Scientific Systems Designs Inc. #BSK6–6) containing room temperature aCSF supplemented with 2.4 mM CaCl_2_ and continuously oxygenated with 95% O_2_ and 5% CO_2_. After a 30 min recovery, slices were treated with either 1 μM PF-719 (Chinglu Pharmaceutical Research) or equal volume of DMSO for 2 h at room temperature. After treatment, slices were immediately homogenized on ice in 100 μl RIPA (EMD Millipore #20–188) with protease and phosphatase inhibitors (Thermo Scientific #1861281) using a polypropylene pellet pestle and spun at 100,000×*g* for 30 min at 4 °C. RIPA-soluble supernatants were boiled in Laemmli sample buffer with 5% β-mercaptoethanol and 1% SDS at 95 °C for 5 min.

### iPSC-derived human cortical neurons

#### Neural induction and terminal differentiation

iPSC-derived human cortical neurons were derived from zero-footprint Gibco Episomal hiPSCs (Gibco #A18945) using a previously described and validated dual SMAD inhibition protocol [40]. hiPSCs were cultured in Essential 8 Flex Medium (Gibco #A2858501) on vitronectin (Gibco #A14700)-coated plates and regularly passaged using Gentle Dissociation Medium (Stemcell Technologies #07174). When confluent, hiPSCs were dissociated using Accutase (Stemcell Technologies #07920) and re-plated at a density of 2*10^5^ cells/well on a vitronectin-coated 12-well plate with 2 μM thiazovivin (Stemcell Technologies #72252) to improve cell-survival. One day after plating (at ~ 75% confluence), Essential 8 Flex Medium was replaced with a neural induction medium [a 1:1 mixture of DMEM/F12 (Gibco #11330) and Neurobasal-A Medium (Gibco #10888022) containing N-2 1:100 (Gibco #17502048), B-27 1:50 (Gibco #17504044), 20 μg/ml insulin (Sigma-Aldrich #I0516), 1 mM L-glutamine (Gibco #25030081), 100 μM MEM Non-Essential Amino Acids (Gibco #11140050), 0.1 mM β-mercaptoethanol (Gibco #21985), 100 nM LDN-193189 (Cayman Chemical #19396), 10 μM SB-431542 (Cayman Chemical #13031) and 2 μM XAV-939 (Tocris #3748)] replaced daily for 12 days. On day 13, cells were dissociated using Accutase and seeded onto 24-well poly-D-lysine plates (Corning #354414) additionally coated with 5 μg/ml laminin (Gibco 23,017,015) in neural induction medium with 2 μM thiazovivin at a density of 4*10^4^ cells/well. Neural induction medium was replaced 2 to 3 days after seeding with terminal neural differentiation medium (Neurobasal-A Medium containing N-2 1:100, B-27 1:50, 1 mM L-glutamine and 30 ng/ml BDNF (Gibco #PHC7074). Cells were maintained in terminal neural differentiation medium, ¾ of which was replenished twice-weekly for more than 120 days. To prevent detachment, terminal neural differentiation medium was supplemented with 2.5 μg/ml laminin once-weekly.

#### hiPSC-derived neuron pharmacology

1 hr prior to treatment, ¾ of medium was replaced with fresh terminal neural differentiation medium. For treatment, cells were administered either PF-719 or DMSO vehicle diluted in terminal neural differentiation medium. For each treatment condition, volumes of DMSO vehicle and DMSO-solubilized PF-719 were kept constant to control for DMSO-induced modulation of cellular signaling events. Neurons were treated for 2 h at 37 °C and, following treatment, immediately harvested on ice in 100 μl/well RIPA (from a 24-well plate) with 1% SDS and protease/phosphatase inhibitors. Samples from adjacent wells were pooled (pooled sample volume, 200 μl from 2 wells), briefly vortexed and spun at 100,000×*g* for 30 min at 4 °C. SDS-soluble supernatants were boiled in Laemmli sample buffer with 5% β-mercaptoethanol and 1% SDS at 95 °C for 5 min.

### Brain tissue collection and processing

9.5–10.5-month-old animals were sacrificed via live decapitation and hemispheres separated medially on ice using a sharp razor blade. For immunohistology, left hemispheres were post-fixed in PBS with 4% paraformaldehyde (PFA) for 48 h at 4 °C. Post-fixed hemispheres were then transferred to PBS with 0.05% sodium azide and stored at 4 °C until sectioning. For biochemistry, hippocampi and cortices were immediately dissected from right hemispheres on ice. To obtain TBS-soluble fractions, hippocampi and cortices were homogenized in 150 μl and 300 μl, respectively, TBS with protease and phosphatase inhibitors using a polypropylene pellet pestle on ice. Homogenates were spun at 100,000×*g* for 30 min at 4 °C. TBS-soluble supernatants were boiled in Laemmli sample buffer with 5% β-mercaptoethanol and 1% SDS at 95 °C for 5 min, while TBS-insoluble hippocampal and cortical pellets were resolubilized on ice in 150 μl and 300 μl, respectively, RIPA with 1% SDS and protease/phosphatase inhibitors. Homogenates were spun at 100,000×*g* for 30 min at 4 °C and SDS-soluble supernatant boiled in Laemmli sample buffer with 5% β-mercaptoethanol and 1% SDS at 95 °C for 5 min.

### Immunoblotting

**Fig. 1:**
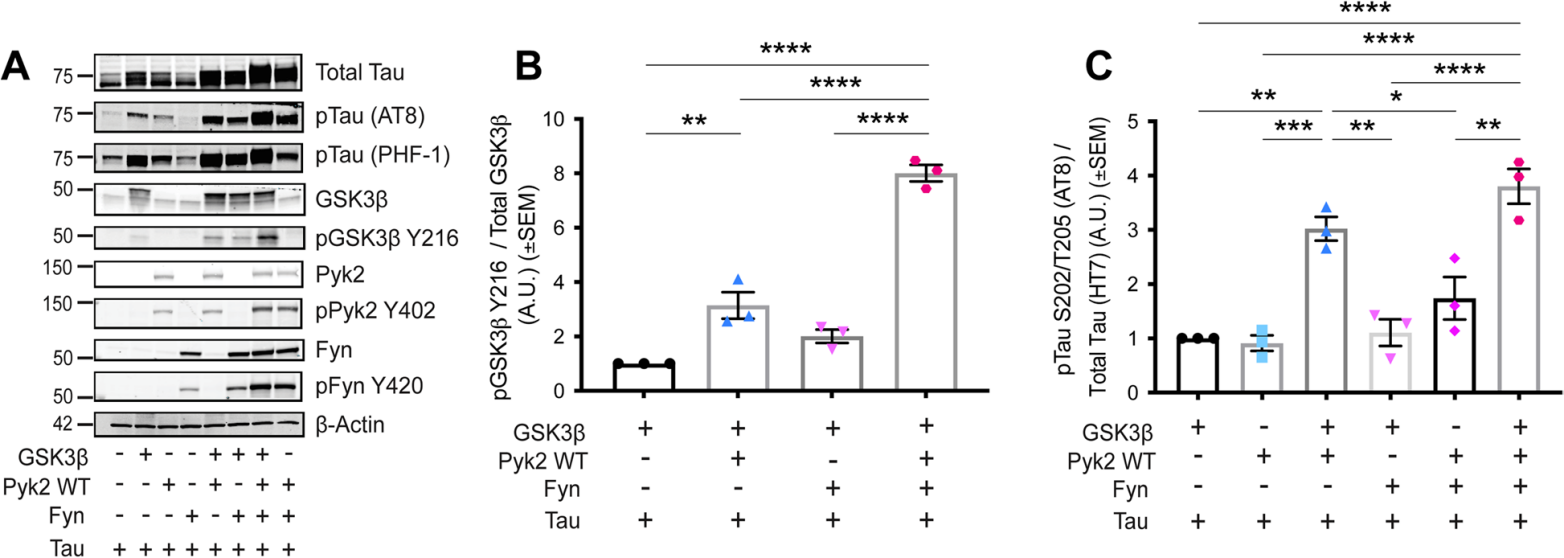
Pyk2 phosphorylates Tau via GSK3β in a Hek293T over-expression system. Hek293T cells were transfected with combinations of the proteins indicated, and lysates separated via SDS-PAGE. Separated lysates were immunoblotted with the antibodies listed. **A**, Representative immunoblot images of transfected Hek293T cells. **B** and **C**, Quantification of **A**. Over-expression of Pyk2 led to a significant increase in the activity of over-expressed GSK3β (pGSK3β Y216 normalized to total GSK3β). This increase was further augmented by the co-transfection of Fyn with GSK3β and Pyk2 (**B**). The phosphorylation of over-expressed Tau at S202/T205 (AT8) normalized to total Tau (HT7) was significantly increased when co-transfected with GSK3β and Pyk2, but not when co-transfected with either kinase alone. No further increase in normalized AT8 signal was observed when Tau, Pyk2 and GSK3β were co-transfected with Fyn. Data are graphed as mean ± SEM, one-way ANOVA with Tukey’s multiple comparisons test, *p < 0.05, **p < 0.01, ***p < 0.001, ****p < 0.0001, *n* = 3

Samples were separated via SDS-PAGE through 4–20% Tris-glycine gels (Bio-Rad #5671095) and transferred onto nitrocellulose membranes (Invitrogen #IB23001) using an iBlot 2 Gel Transfer Device (Invitrogen #IB21001). Loaded sample volumes were normalized to total protein concentration via BCA protein assay (Thermo Scientific #23225). Nitrocellulose membranes were blocked while rocking at room temperature for 1 h (Rockland #MB-070-010TF) and incubated with blocking buffer containing primary antibodies overnight at 4 °C. The following antibodies were employed for immunoblotting: anti-Tau (HT7) (Thermo Fisher Scientific #MN1000, 1:1000, RRID:AB_2314654), anti-pTau S202/T205 (AT8) (Thermo Fisher Scientific #MN1020, 1:1000, RRID:AB_223647), anti-pTau S396/S404 (PHF-1) (Peter Davies personal request, 1:1000, RRID:AB_2315150), anti-pTau S199/S202 (Thermo Fisher Scientific #44-768G, 1:1000, RRID:AB_2533749), anti-pTau T181 (AT270) (Thermo Fisher Scientific #MN1050, 1:1000, RRID:AB_223651), anti-pTau S262 (Thermo Fisher Scientific #44-750G, 1:1000, RRID:AB_2533743), anti-Pyk2 (Cell Signaling Technology #3480, 1:1000, RRID:AB_2174093), anti-pPyk2 Y402 (Cell Signaling Technology #3291, 1:1000, RRID:AB_2300530), anti-GSK3β (Cell Signaling Technology #9315, 1:1000, RRID:AB_490890), anti-GSK3β Y216/pGSK3α Y279 (Abcam #ab68476, 1:1000, RRID:AB_10013745), anti-GSK3β S9 (Cell Signaling Technology #9336, 1:1000, RRID:AB_331405), anti-Fyn (Cell Signaling Technology #4023, 1:1000, RRID:AB_10698604), anti-pSrc Family Y216 (Cell Signaling Technology #6943, 1:1000, RRID:AB_10013641), anti-PSD-95 (Cell Signaling Technology #36233, 1:1000, RRID:AB_2721262), anti-C1q (Abcam #ab182451, 1:1000, RRID:AB_2732849), anti-p38 MAPK (Cell Signaling Technology #9212, 1:1000, RRID:AB_330713), anti-pp38 MAPK T180/Y182 (Cell Signaling Technology #4511, 1:1000, RRID:AB_2139682), anti-LKB1 (Cell Signaling Technology #3047, 1:1000, RRID:AB_2198327), anti-pLKB1 S428 (Cell Signaling Technology #3482, 1:1000, RRID:AB_2198321), anti-MAPK1 (Cell Signaling Technology #4696, RRID:AB_390780), anti-pMAPK1 T185/Y187 (Cell Signaling Technology #9101, 1:1000, RRID:AB_331646) and anti-β-Actin (Cell Signaling Technology #3700, 1:10,000, RRID:AB_2242334). For experiments shown in Fig. 1, all primary antibodies were the same as above accept for the following: anti-GSK3β (Cell Signaling Technology #12456, 1:2000, RRID:AB_2636978), anti-GSK3β Y216/pGSK3α Y279 (Abcam #ab52188, 1:1000, RRID:AB_880261), anti-Pyk2 (Cell Signaling Technology #3292, 1:1000, RRID:AB_2174097) and anti-β-Actin (Cell Signaling Technology #8457, 1:1000, RRID:AB_10950489). After primary antibody incubation, membranes were washed (3 X 5-min in TBST) and incubated with appropriate secondary antibodies for 1 h at room temperature. The following secondary antibodies were used for immunoblotting: anti-mouse IRDye 800CW (LI-COR Biosciences #926–32,212, 1:20,000, RRID:AB_621847) and α-rabbit IRDye 680CW (LI-COR Biosciences #926–68,023, 1:20,000, RRID:AB_10706167). After final washes (5 X 5-min in TBST), immunoblots were scanned with a LI-COR Odyssey infrared imaging system and protein bands quantified using LI-COR Image Studio Lite software, version 5.2.5 (RRID:SCR_013715).

### Immunohistology

#### Immunofluorescence

Brains post-fixed in 4% PFA for 48 h were vibratome sectioned into 40 μm, free-floating sections, washed in PBS with 0.05% sodium azide and blocked with PBS containing 0.1% Triton X-100 (American Bio #AB02025) and 1% BSA for 1 h at room temperature. Spinal cord lumbar enlargements were post-fixed and, following embedment in gelatin, sectioned and blocked as described above. Sections were incubated in primary antibody diluted in PBS with 0.1% Triton X-100 and 1% BSA for 48 h at 4 °C. The following primary antibodies were used for immunohistology: anti-Pyk2 (Santa Cruz Biotechnology #SC-1515, 1:250, RRID:AB_632286), anti-Tau (Agilent #A0024, 1:500, RRID:AB_10013724), anti-pTau S202/T205 (AT8) (Thermo Fisher Scientific #MN1020, 1:1500, RRID:AB_223647), anti-pTau S199/S202 (Thermo Fisher Scientific #44-768G, 1:100, RRID:AB_2533749), anti-NeuN (Abcam #ab104225, 1:500, RRID:AB_10711153), anti-GFAP (Abcam #ab4674, 1:500, RRID:AB_304558), anti-Iba1 (FUJIFILM Wako Shibayagi #019–19,741, 1:250, RRID:AB_839504), anti-CD68 (Bio-Rad #MCA1957, 1:500, RRID:AB_322219), anti-PSD-95 (Thermo Fisher Scientific #51–6900, 1:250, RRID:AB_2533914) and anti-C1q (Abcam #ab182451, 1:1000, RRID:AB_2732849). Anti-PSD-95 immunolabeling required an antigen retrieval step prior to primary antibody incubation. For antigen retrieval, sections were transferred to PBS with 1% SDS and heated at 90 °C for 5 min. Following primary antibody incubation, sections were washed (3 X in PBS) and incubated in appropriate secondary antibodies diluted in PBS with 0.1% Triton X-100 and 1% BSA overnight at 4 °C. The following Alexa Fluor secondary antibodies were employed: anti-goat 488 (Thermo Fisher Scientific #A11055, 1:500, RRID:AB_2534102), anti-mouse 488 (Thermo Fisher Scientific #A-21202, 1:500, RRID:AB_141607), anti-rabbit 568 (Thermo Fisher Scientific #A10042, 1:500, RRID:AB_2534017), anti-rabbit 488 (Thermo Fisher Scientific #A-11008, 1:500, RRID:AB_143165), anti-chicken 647 (Thermo Fisher Scientific #A32933, 1:500, RRID:AB_2762845) and anti-rat 488 (Thermo Fisher Scientific #A-21208, 1:500, RRID:AB_2535794). To minimize lipofuscin autofluorescence, sections were washed after secondary antibody incubation (3 X in PBS), dipped briefly in ddH_2_O and incubated in ammonium acetate with 10 mM CuSO_4_ for 15 min at room temperature. Samples were briefly returned to ddH_2_O, washed in PBS and mounted onto glass slides using Vectashield mounting medium with DAPI (Vector #H-1200).

#### Cresyl violet staining

Post-fixed brain sections were obtained as described above and stained in filtered, pre-warmed 0.1% cresyl violet solution for 10 min. Sections were then washed in ddH_2_O for 3 min and de-stained in 95 and 100% ethanol for 10 and 5 min, respectively. Sections were submerged in fresh 100% ethanol for an additional 5 min, placed in xylene for 5 min, removed and placed in fresh xylene for an additional 5 min. Sections were left in xylene overnight and then mounted to glass slides with Cytoseal 60 (Thermo Fisher Scientific #8310–04).

### Imaging and immunohistological analysis

#### Image acquisition

Images of Pyk2, total Tau, phospho-Tau, NeuN, Iba1, CD68 and C1q-immunolabeled sections were captured using a Leica SP8 confocal microscope. Pyk2-immunolabeled sections were imaged using a 10X 0.4 numerical aperture air-objective lens. 4 image slices were acquired throughout the thickness of each section and z-stack compressed via maximum orthogonal projection. 12 contiguous, tiled images (3 × 4) were stitched together to image the hippocampus and cortex. Tau-immunolabeled sections were imaged with a 10X 0.4 numerical aperture air-objective lens. 20 image slices were acquired throughout the entire thickness of each section and z-stack compressed via maximum orthogonal projection. NeuN- and AT8-immunolabled spinal cord sections were imaged with a 10X 0.4 numerical aperture air-objective lens. 10 image slices were acquired throughout the thickness of each section and z-stack compressed via maximum orthogonal projection. To image the entire spinal cord lumbar enragement, 6 contiguous, tiled images (2 × 3) were stitched together. Iba1 and CD68-immunolabed sections were imaged using a 20X 0.75 numerical aperture air objective and 15 image slices, acquired throughout the thickness of each section, z-stack compressed via maximum orthogonal projection. To image the entire hippocampus, 4 contiguous, tiled images (1 × 4) were stitched together. For C1q imaging, sections were imaged with a 63X 1.4 numerical aperture oil-immersion objective and 5 image slices (1 μm apart) were z-stack compressed via maximum orthogonal projection. For PSD-95 and GFAP imaging, section images were captured using a Zeiss LSM 800 confocal microscope. PSD-95-immunolabeled sections were imaged using a 63X 1.4 numerical aperture oil-immersion objective at the z-level of greatest immunofluorescence for each section. GFAP-labeled sections were imaged with a 20X 0.8 numerical aperture air objective and 10 image slices taken throughout the thickness of the section were z-stack projected via maximum orthogonal projection. To image the entire dentate gyrus, 3 × 5 tiled images were stitched together. For all image acquisition, pinholes were set to 1 AU. Cresyl violet-stained sections were scanned using an Aperio scanner (Aperio CS2, Leica Biosystems).

#### Image analysis

Image analysis was conducted using CellProfiler Image Analysis software, version 3.1.8 (RRID:SCR_007358). Macros to identify positive immunofluorescence signal for cell bodies or synaptic puncta were developed and applied uniformly for each immunolabeled epitope. Hippocampal cell layer thickness of cresyl violet-stained sections was determined using Aperio ImageScope, version 12.4.3.5008 (RRID:SCR_020993). All images were acquired, processed and analyzed by an experimenter blinded to animal genotype.

### Behavioral assays

Behavioral assays were administered in the following order: rotarod, wire hang and Morris Water maze (MWM). Noldus CatWalk XT gait analysis was administered to a separate cohort of animals naïve to rotarod, wire hang and MWM. All animals were 9–10 months old at the time of behavioral testing. Exclusion criteria, described below, were applied independently to each assay. Animal handling and analyses (including application of exclusion criteria) were completed by an experimenter blinded to animal genotype. In order to habituate animals to experimenter handling, mice were handled for at least 2 min over 4 days prior to the first behavioral assay. For each assay, mice were habituated to the testing room for 1 h prior to behavioral testing.

#### Rotarod

Animals were positioned on the drum of a 4-lane Rotarod device (Economex, Columbus Instruments) set to accelerate 0.1 rotations/min/sec until reaching a top speed of 4 rpm. Trials began at the start of drum rotation. Latency to fall onto a foam pad placed beneath the spinning drum was recorded across five trials with 1 min inter-trial rest periods.

#### Wire hang

Animals were placed in the center of a wire grid (wire spacing, 1 cm × 1 cm) which, at the beginning of each trial, was inverted over a foam pad. The latency to fall from the grid was recorded (maximum trial duration, 120 s) across two trials with a 1 min inter-trial rest period.

#### MWM

Testing took place over 5 consecutive days, with acquisition trials taking place over the first 3 days and the probe and visible platform trials taking place on days 4 and 5, respectively. Mice were swum in an open pool ~ 1 m in diameter adorned with distinct visual cues (white poster board marked with black tape) distributed evenly along the pool perimeter. Water temperature was set to 25 °C at the beginning of each testing day. During acquisition, a clear plexiglass platform submerged 1 cm below water level was placed in the center of the target quadrant where it remained for the duration of the acquisition trials. Acquisition trials took place over 6 sessions, two sessions per day, with each session consisting of 4 trials and 1 min inter-trial rest periods. For each trial, mice were placed (facing away from the pool’s center) into the pool at one of four drop zones, the orders of which were varied across each session. The time required to locate the submerged platform was recorded for each trial using Panlab SMART video tracking software, version 2.5.21 (RRID:SCR_002852). A trial was considered successfully completed only if the animal spent 1 s on the platform. Animals unable to locate the submerged platform within the 60 s maximum trial period were gently guided to the platform and allowed to rest for 10 s. 24 h after the final acquisition trial, animals were reintroduced to the pool with the submerged platform removed. During the single probe trial, animals were placed in the zone farthest from the target quadrant and the time spent in the target quadrant recorded (maximum trial duration, 60 s). To rule out visual impairment as a potential confound, the latency required to reach a visible platform placed in the center of the pool was determined. Visible platform trials were administered until latencies stabilized (defined as a maximum range in latency of 10 s across 3 consecutive trials) over a maximum number of 15 trials, and the latencies for the final 3 trials were averaged. Animals that were unable to locate the visible platform (1 PS19^0/+^ and 1 PS19^0/+^;Pyk2^−/−^ mouse), were statistical outliers (1 Pyk2^−/−^ mouse) or whose latencies failed to stabilize over 15 trials (1 Pyk2^−/−^ mouse) were excluded from all MWM analyses.

#### Noldus CatWalk XT

Prior to behavioral testing, animals were habituated and trained to the CatWalk by placing their home-cage beneath a platform located at the target end of the CatWalk unit. The home-cage was accessible to the animals through an aperture in the platform. Animals were placed onto the target end platform and permitted to explore until they attempted to enter their home-cage through the platform aperture. After 3 successful attempts, a housing-unit was placed over the aperture bridging the end of the CatWalk and the home-cage entrance. Testing began after 3 successful attempts to enter the housing unit from the target end of the CatWalk. Animals were placed at the far end of the CatWalk unit and gait parameters recorded during each successful transverse of the ~ 1 m long platform track in either direction. Trials were repeated until animals achieved 3 successful runs (maximum run duration, 10 s; maximum run variation, 75%). Animals unable to ambulate across the platform track were excluded from analysis.

### Label-free quantitative proteomics

#### Sample preparation

P2’ crude synaptosomal pellets from mouse brain (4 biological replicates / genotype) were homogenized by sonication in a buffer containing urea (8 M), ammonium bicarbonate (0.4 M), and protease (Pierce #87786 at 1% of lysis buffer) and phosphatase inhibitor cocktails (Pierce #78420 at 2.5% of lysis buffer). Samples were then centrifuged to pellet cellular debris and supernatant collected for downstream global proteomics sample preparation with slight modification from a previously published protocol [41]. Briefly, proteins were first extracted using cold (− 20 °C) acetone. A 1:4 ratio of protein solution to cold acetone was incubated for 1 h at − 20 °C, then centrifuged at 15,000×*g* to pellet out the protein precipitate. Protein pellets were reconstituted in a final solution containing 2 M urea and 25 mM ammonium bicarbonate. Cysteines were reduced with dithiothreitol (DTT) at 37 °C for 20 min, cooled and then alkylated with iodoacetamide (IAM) at room temperature in the dark for 20 min. Dual enzymatic digestion was carried out first with LysC at 37 °C for 4 h and subsequently with Trypsin overnight at 37 °C. Digestion was quenched with 0.5% formic acid. Samples were desalted using MiniSpin SPE columns (The Nest Group #HMM S18V) and dried via SpeedVac (Thermo Scientific SAVANT RVT-4104). Pellets were dissolved in a solution containing 70 mM L-glutamic acid, 65% ACN and 2% TFA in water (loading/conditioning buffer for TopTip). Samples were then subjected to titanium dioxide (TiO_2_) phospho-peptide enrichment using TopTip (Glygen #TT2TIO). Phospho-peptide enrichment was carried out according to manufacturing specification with the exception of the initial loading/conditioning buffer indicated above. Flow Through peptide eluate (FT, non-bound) was collected and stored at − 80 °C for mass spectrometry analysis of total proteins. Enriched phospho-peptides (EN, bound) were eluted from each TopTip in three aliquots of 30 μL 28% high-purity ammonium hydroxide. The eluted fraction was dried and re-dried with 2 × 30 μL water by SpeedVac. Enriched fractions were dissolved in 10 μL of 70% formic acid and 30 μL of 50 mM sodium phosphate. Peptide concentrations were determined by NanoDrop spectrophotometer (Thermo Scientific NanoDrop 2000) to load 0.3 μg / 5 μL of each sample for analysis (3 injections / biological replicate). For WT vs Pyk2^−/−^ analysis, LC-MS/MS was conducted using an Orbitrap Fusion LC-MS/MS mass spectrometer equipped with a Waters nanoACQUITY Ultra Performance Liquid Chromatography (UPLC) System. For the PS19^0/+^ vs PS19^0/+^;Pyk2^−/−^ analysis, LC-MS/MS was conducted using a Q Exactive HF-X Quadrupole-Orbitrap MS system.

#### Proteomics data analysis

Online chromatographic separation was conducted as described previously [42]. Peaks were selected and the generated peak list files were used for phospho-peptide identification using SEQUEST search algorithm in Proteome Discoverer, version 2.2 (RRID:SCR_014477). Searches were conducted against the SwissProt Protein Database (Version SwissProt_2017_01, *Mus musculus*) (RRID:SCR_017486). Search parameters included: fragment ion mass tolerance of 0.020 Da; parent ion tolerance of 10.0 pp.; strict trypsin fragments (enzyme cleavage after the C-terminus of Lysine or Arginine, but not if followed by Proline); variable modification of phospho- Ser, Thr, and Tyr; oxidation of Met; deamidation of Asn and Gln; and carbamidomethlyation of Cys. A decoy search (based on the reverse sequence search) was performed to estimate the false discovery rate (FDR), with a setting of acceptable protein ID having an FDR of < 1%. A protein was considered to be positively identified if there were two or more significantly labeled unique peptides. Scaffold Proteome Software, version 4.8.6 (RRID:SCR_014345) was used to obtain quantitative abundance values for comparison between WT and Pyk2^−/−^ genotypes. Abundance values for phospho-enriched samples were normalized to total (FT) values. Proteins significantly differentiated between genotypes were identified using two-tailed *t*-test (*p* < 0.05). Two-tailed *t*-tests were conducted and z-scores determined using Microsoft Excel software, version 16.16.27 (RRID:SCR_016137). Enrichment of Gene Ontology terms amongst lists of DEPs were assessed using ClueGO in Cytoscape ([43], RRID: SCR_005748). A protein-protein association network between significantly differentially regulated phospho-proteins was obtained using STRING ([44], RRID:SCR_005223).

### Synaptosomal fractionation

Animals were sacrificed and brain tissue dissected on ice as described above. Hippocampi and cortices from a single hemisphere were homogenized in 200 μl and 400 μl, respectively, Syn-PER Reagent (Thermo Scientific #87793) with protease and phosphatase inhibitors using a polypropylene pellet pestle. Homogenates were centrifuged at 1200×*g* for 10 min at 4 °C. Supernatants were collected and spun again at 15,000×*g* for 20 min at 4 °C. The crude synaptosomal (P2’) pellets were resolubilized in RIPA with 1% SDS and boiled in Laemmli sample buffer with 5% β-mercaptoethanol and 1% SDS at 95 °C for 5 min.

### Experimental design and statistical analysis

One-way ANOVA with post hoc Tukey’s multiple comparisons tests, One-way ANOVA with post hoc Dunnett’s multiple comparisons tests, Log-rank (Mantel-Cox) test and unpaired two-tailed *t*-tests were performed using GraphPad Prism software, versions 8 and 9 (RRID:SCR_002798). Repeated measures ANOVA with post hoc Tukey HSD multiple comparisons tests were performed using IBM SPSS Statics software, version 26 (RRID:SCR_019096). For the MWM visible platform test, statistical outliers were identified using the ROUT method (Q = 1%) in GraphPad Prism. Group means ± SEM and samples sizes (*n*) are reported in each figure legend. Data were considered to be statistically significant if *p* < 0.05. For all figures, all statistically significant group differences are labeled. For any given group comparison, the lack of any indication of significant difference implies lack of significance by the applied statistical test.

## Results

### Pyk2 phosphorylates tau via GSK3β in an in vitro over-expression system

The ability of GSK3β to phosphorylate pathophysiologically-relevant resides of Tau is well-documented [21–26], and Pyk2 has been shown activate GSK3β through the phosphorylation of Y216 in GSK3β’s activation loop [27–29]. Although it has previously been demonstrated that Pyk2 can directly phosphorylate Tau at Y18 [31], whether Pyk2 can augment GSK3β’s ability to phosphorylate Tau at disease-relevant residues has not been clarified. We over-expressed combinations of GSK3β, Pyk2, Tau and Fyn in Hek293T cells and measured the phosphorylation of GSK3β at Y216 and Tau at S202/T205 (AT8) (Fig. 1A–C). Pyk2 co-transfection with GSK3β led to a significant increase in GSK3β activation, and this activation was further augmented with the addition of Fyn, a kinase known to synergistically co-activate Pyk2 [10, 45–47] (Fig. 1A, B). Over-expression of GSK3β and Pyk2 together led to a significant increase in Tau phosphorylation at S202/T205 compared Tau co-transfected with either kinase alone (Fig. 1A, C). Over-expression of GSK3β, Pyk2 and Fyn together also led to robust Tau phosphorylation at S202/T205, however this signal failed to reach a level significantly higher than that seen with both GSK3β and Pyk2, suggesting that a ceiling to GSK3β-mediated S202/T205 phosphorylation may have been achieved in this system.

### Basal levels of Pyk2 activity suppress tau phosphorylation in neurons

**Fig. 2:**
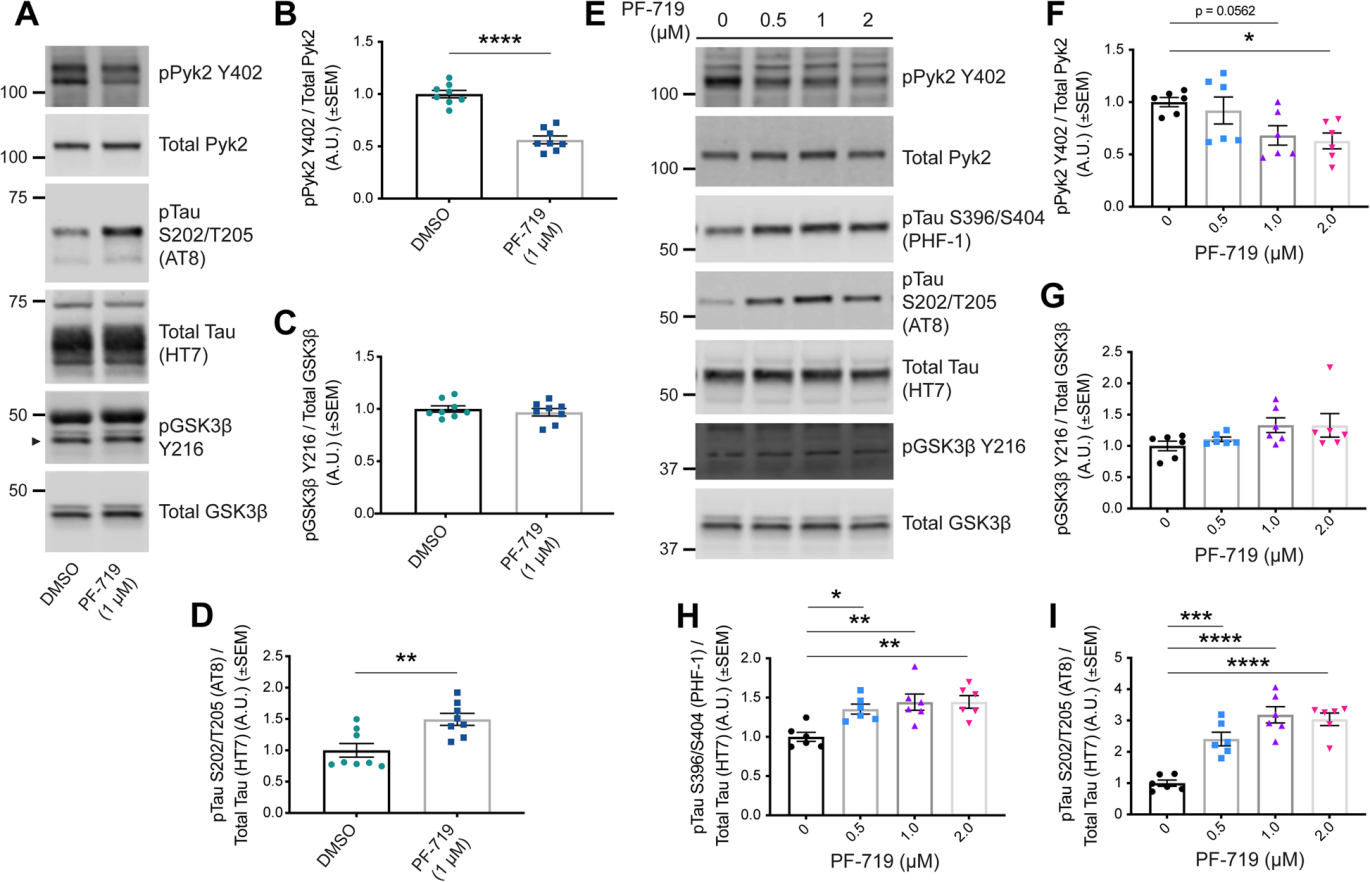
Pharmacological inhibition of Pyk2 increases Tau phosphorylation independent of changes in GSK3β activity. **A**–**D**, Acute hippocampal slices (thickness, 400 μm) from 4.5–5.5-month-old PS19^0/+^ animals were treated with 1 μM Pyk2 inhibitor (PF-719) for 2 h in oxygenated artificial CSF at room temperature and were homogenized in RIPA immediately following treatment. RIPA-soluble lysates were separated by SDS-PAGE and immunoblotted with the antibodies indicated. **A**, Representative immunoblot images of lysates from PF-719-treated acute hippocampal slices. Arrowhead indicates pGSK3β Y216. **B**–**D**, Quantification of **A**. PF-719 treatment successfully inhibited Pyk2 activity (pPyk2 Y402 normalized to total Pyk2). Pyk2 inhibition significantly increased the phosphorylation Tau at S202/T205 (AT8) normalized to total Tau (HT7) (**D**) while GSK3β activity (pGSK3β Y216 normalized to total GSK3β) remained unchanged (**C**). Data are graphed as mean ± SEM, unpaired two-tailed *t*-test, **p < 0.01, ****p < 0.0001, *n* = 8. **E**–**I**, iPSC-derived human cortical neurons (90–100 days post terminal differentiation) were treated with PF-719 at indicated concentrations for 2 h at 37 °C and, immediately following treatment, homogenized in RIPA containing 1% SDS. Lysates were separated by SDS-PAGE and immunoblotted with the antibodies listed. **E**, Representative immunoblot images of lysates from PF-719-treated iPSC-derived human cortical neurons. **F**–**I**, Quantification of **E**. PF-719 treatment significantly inhibited Pyk2 activity (pPyk2 Y402 normalized to total Pyk2) (**F**), while no changes in GSK3β activity (pGSK3β Y216 normalized to total GSK3β) were observed at any concentration of PF-719 (**G**). Pyk2 inhibition resulted in increased levels of Tau phosphorylation at S396/S404 (PHF-1) normalized to total Tau (HT7) (**H**) and S202/T205 (AT8) normalized to total Tau (HT7) (**I**) at every concentration of PF-719 administered. Data are graphed as mean ± SEM, one-way ANOVA with Dunnett’s multiple comparisons test, *p < 0.05, **p < 0.01, ***p < 0.001, ****p < 0.0001, *n* = 6

**Fig. 3:**
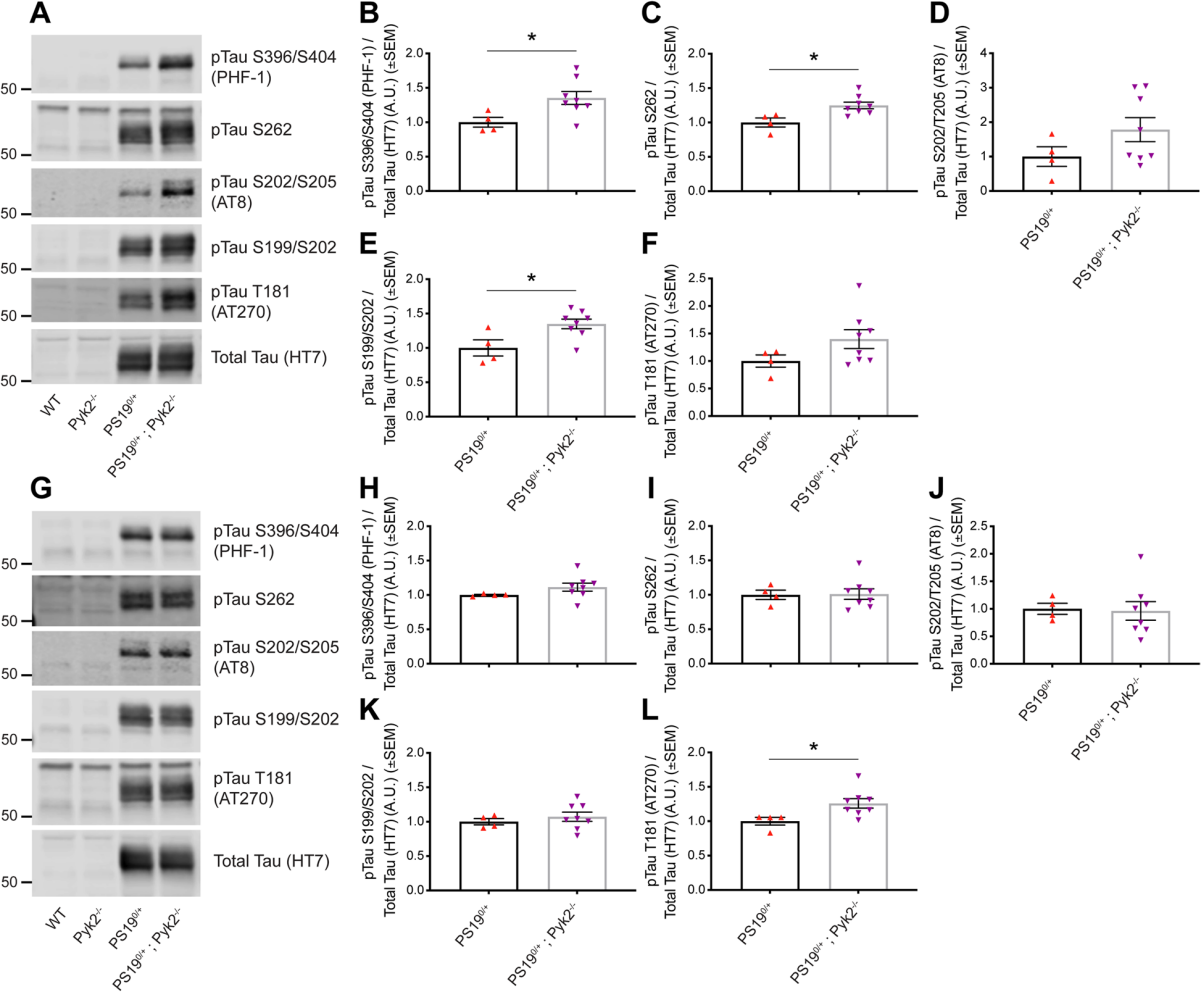
Pyk2 expression suppresses Tau phosphorylation in a PS19^0/+^ animal model of tauopathy. **A**–**L**, TBS-insoluble, SDS-soluble hippocampal (**A**–**F**) and cortical (**G**–**L**) lysates from 9.5–10.5-month-old WT, Pyk2^−/−^, PS19^0/+^ and PS19^0/+^;Pyk2^−/−^ animals were separated by SDS-PAGE and immunoblotted with antibodies against multiple pathophysiologically-relevant phospho-Tau residues as well as total Tau. **A**, Representative immunoblot images of TBS-insoluble, SDS-soluble hippocampal Tau. **B**–**F**, Quantification of protein levels by densitometric analysis reveals significantly greater phosphorylation of hippocampal Tau in lysates from PS19^0/+^;Pyk2^−/−^ animals at pTau S396/S404 (PHF-1) (**B**), pTau S262 (**C**) and pTau S199/S202 (**E**) compared to lysates from PS19^0/+^ animals. All data are normalized total (HT7) levels of hippocampal Tau. Data are graphed as mean ± SEM, unpaired two-tailed *t*-test, *p < 0.05, *n* = 4–8 mice. **G**, Representative immunoblot images of TBS-insoluble, SDS-soluble cortical Tau. **H**–**L**, Quantification of protein levels by densitometric analysis reveals significantly greater phosphorylation of cortical Tau in lysates from PS19^0/+^;Pyk2^−/−^ animals at pTau S262 (**L**) compared to those from PS19^0/+^ animals. All data are normalized to total (HT7) levels cortical Tau. Data are graphed as mean ± SEM, unpaired two-tailed *t*-test, *p < 0.05, *n* = 4–8 mice

In order to confirm whether Pyk2 activity positively correlates with Tau phosphorylation at physiological levels of expression in neurons ex vivo, we conducted an acute brain slice assay in which we pharmacologically inhibited Pyk2 in mouse hippocampal slices with the selective Pyk2 inhibitor PF-719 (Fig. 2A–D). Since basal levels of AT8 Tau phosphorylation were undetectable in WT mice (Fig. 3A, G), we used tissue from transgenic PS19^0/+^ mice that express a mutant form of human Tau (MAPT P301S) associated with FTD and are a well-described animal model of tauopathy [48, 49]. Unexpectedly, inhibition of Pyk2 with 1 μM PF-719 resulted in an increase of Tau phosphorylation at S202/T205 (Fig. 2A, B, D). Importantly, this increase in Tau phosphorylation occurred independently of any changes in GSK3β activity (Fig. 2A, C), suggesting that basal levels of Pyk2 activity suppress Tau phosphorylation through a GSK3β-independent mechanism.

To confirm these results in a second neuronal system, we pharmacologically inhibited Pyk2 in mature iPSC-derived human cortical neurons differentiated from iPSCs via dual SMAD inhibition and patterned into forebrain cortical neurons via Wnt inhibition. This well-described and previously validated method of obtaining cortical neurons from iPSCs has the benefit of recapitulating the differentiation of cortical neurons in the developing human brain without relying on the induced overexpression of transgenic transcription factors such as Ngn2 [40]. While Pyk2 inhibition failed to result in any measurable modulation of GSK3β activity in these neurons, the inhibition of basal Pyk2 activity with PF-719 led to significant increases in Tau phosphorylation at two pathophysiologically-relevant epitopes of Tau, S396/S404 (PHF-1) and S202/T205 (AT8) (Fig. 2E–I**)**. Together, these results suggest that while Pyk2 can phosphorylate Tau through GSK3β when over-expressed at supra-physiological stoichiometries, basal levels of neuronal Pyk2 activity suppress Tau phosphorylation through a mechanism independent of measurable changes in GSK3β activity.

### Pyk2 expression is protective against tau phosphorylation and pathology in vivo

To determine whether Pyk2 expression is protective against Tau phosphorylation in an in vivo mammalian system, we again employed PS19^0/+^ transgenic mice, this time interbred with a Pyk2^−/−^ line. To assess the influence of Pyk2 genetic deletion on Tau phosphorylation biochemically, we obtained TBS-insoluble, SDS-soluble fractions from both hippocampi and cortices of 9.5–10.5-month-old WT, Pyk2^−/−^, PS19^0/+^ and PS19^0/+^;Pyk2^−/−^ animals (Fig. 3A–L). While there were no detectable levels of TBS-insoluble, SDS-soluble Tau phosphorylated at any disease-relevant epitope analyzed biochemically in WT or Pyk2^−/−^ mice, PS19^0/+^;Pyk2^−/−^ animals demonstrated a significantly higher level of hippocampal Tau phosphorylation at S393/S404 (PHF-1), S262 and S199/S202 compared to PS19^0/+^ mice (Fig. 3A–F). Of phospho-Tau epitopes analyzed in the cortex, only pTau T181 (AT270) demonstrated higher phosphorylation in PS19^0/+^;Pyk2^−/−^ compared to PS19^0/+^ animals, suggesting that Pyk2’s influence on regulating Tau phosphorylation may be greater in hippocampus compared to cortex (Fig. 3G–L). In support of a region-specific influence of Pyk2 in suppressing Tau phosphorylation, we observed significantly greater Pyk2 expression in the hippocampus compared to cortex both immunohistologically and biochemically (Fig. S1A–C). Pyk2 activation was also significantly higher in WT and PS19^0/+^ hippocampus compared to cortex, while Tau-induced activation of Pyk2 in PS19^0/+^ animals was restricted to the hippocampus (Fig. S1D). Higher levels of expression and activity of Pyk2 in the hippocampus suggest that its genetic deletion from PS19^0/+^ animals would likely have a greater magnitude of effect on Tau phosphorylation in this region compared to cortex.

**Fig. 4:**
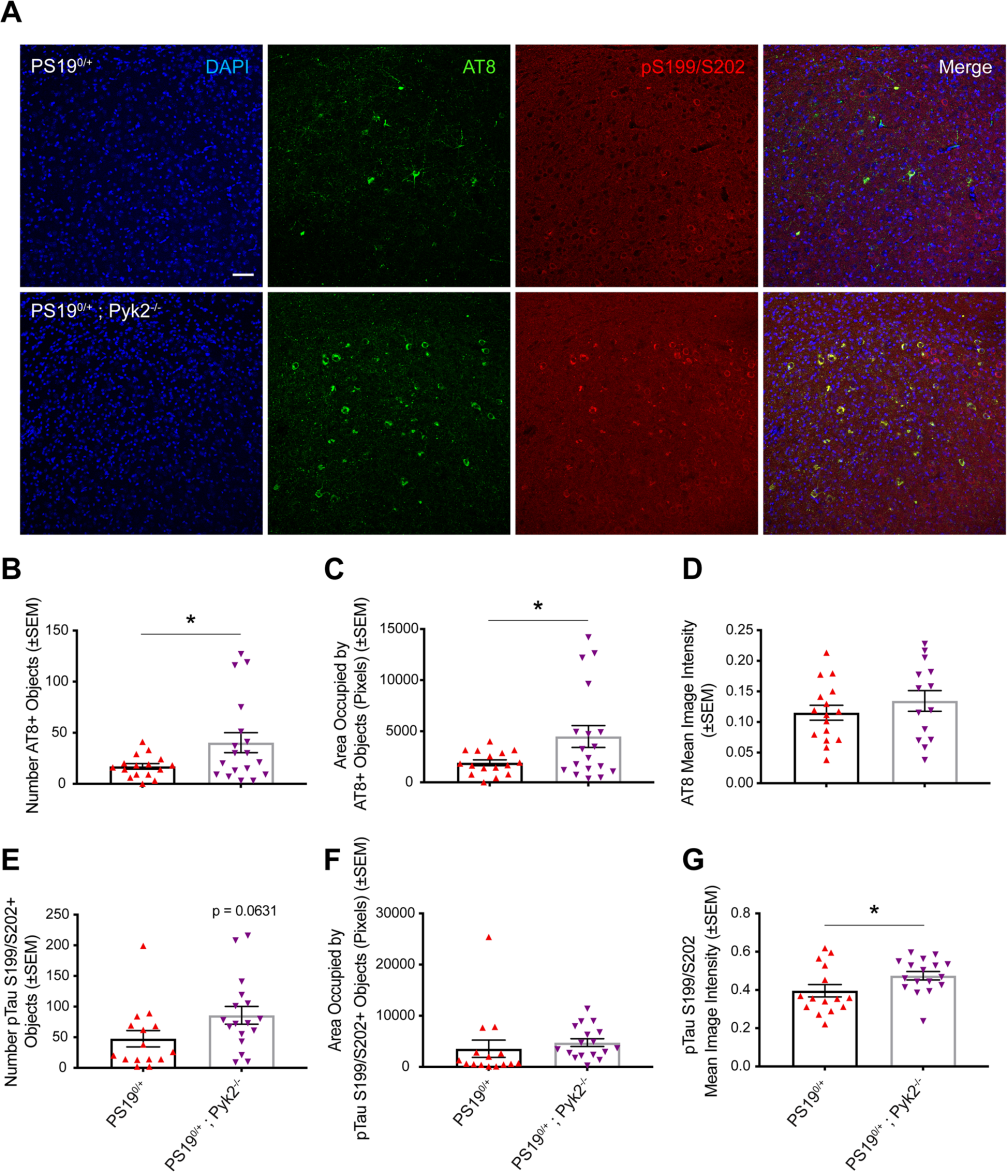
Pyk2 expression is protective against Tau pathology in PS19^0/+^ mice. **A**, Representative immunofluorescent images of DAPI, pTau S202/T205 (AT8) and pTau S199/S202 immunoreactivity in amygdala of 9.5–10.5-month-old WT, Pyk2^−/−^, PS19^0/+^ and PS19^0/+^;Pyk2^−/−^ animals. Scale bar, 50 μm. **B**–**D**, Quantification of amygdalar pTau S202/T205 (AT8) immunoreactivity reveals significant increases in the number of AT8-positive cell-bodies (objects) (**B**) as well as in the area occupied by those objects (**C**) in PS19^0/+^;Pyk2^−/−^ compared to PS19^0/+^ animals. Data are graphed as mean ± SEM, unpaired two-tailed *t*-test, *p < 0.05, *n* = 14–16 mice. **E**–**G**, Quantification of amygdalar pTau S199/S202 immunoreactivity reveals a significant increase in pTau S199/S202 mean image intensity (**G**) in PS19^0/+^;Pyk2^−/−^ compared to PS19^0/+^ animals. Data are graphed as mean ± SEM, unpaired two-tailed *t*-test, *p < 0.05, *n* = 15–18 mice

The ability of Pyk2 expression to suppress Tau phosphorylation, however, extends beyond hippocampus and cortex. Histological analysis of 9.5–10.5-month-old PS19^0/+^ and PS19^0/+^;Pyk2^−/−^ amygdala also revealed significant increases in immunofluorescent signal of pTau S202/T205 (AT8) in both the number of AT8-positive cell bodies, as well as the area occupied by those cell bodies, and pTau S199/S202 by mean image intensity of immunofluorescent signal (Fig. 4A–G). While sexual dimorphism with respect to severity of Tau pathology has been previously reported in PS19 transgenic mice, we observed no differences in amygdalar Tau pathology between PS19^0/+^ and PS19^0/+^;Pyk2^−/−^ animals by any measure when segregated by sex (Fig. S2) [50]. No changes were observed in total Tau immunofluorescence between PS19^0/+^ and PS19^0/+^;Pyk2^−/−^ animals, suggesting that Pyk2’s ability to suppress Tau pathology is specific to Tau phosphorylation rather than total Tau expression (Fig. S3). Taken together, these results provide both biochemical and histological evidence of Pyk2’s role in suppressing Tau phosphorylation in a well-described animal model of tauopathy in vivo.

### Pyk2 expression is protective against tau-induced early death and memory impairment in PS19 transgenic mice

**Fig. 5:**
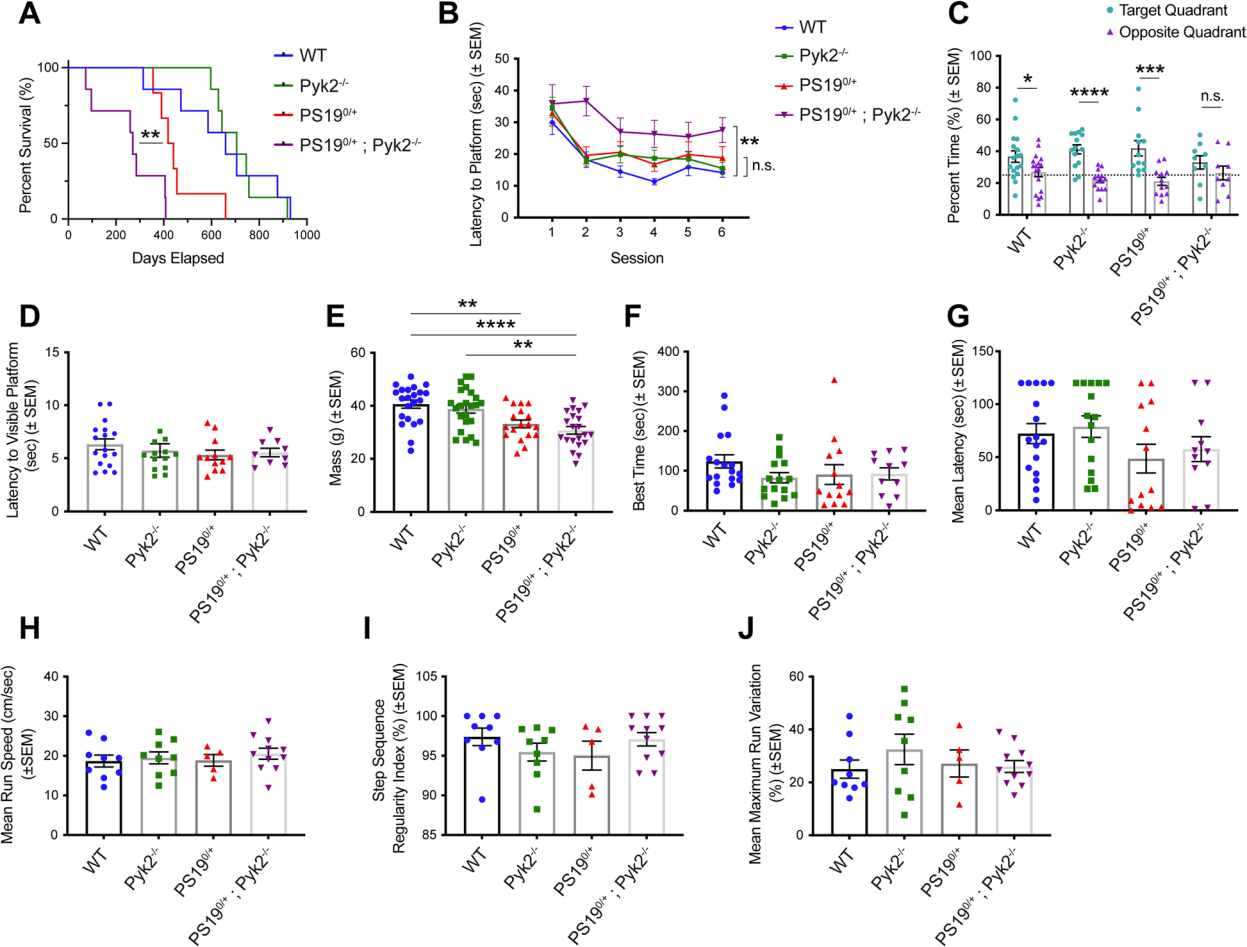
Pyk2 expression is protective against Tau-mediated early death and spatial memory impairment in PS19^0/+^ animals. **A**, Kaplan-Meier survival curve of WT, Pyk2^−/−^, PS19^0/+^ and PS19^0/+^;Pyk2^−/−^ animals. Survivorship of PS19^0/+^;Pyk2^−/−^ mice (median survival, 270 days) is significantly reduced compared to PS19^0/+^ animals (median survival, 429.5 days). Log-rank (Mantel-Cox) test, **p = 0.0085, *n* = 6–7 mice. **B**–**D**, Spatial memory of 9–10-month-old mice was assessed using the MWM test. **B**, Latency is defined as the time required to reach a hidden platform across 6 acquisition sessions of 4 trials each. Across the final 4 acquisition sessions, PS19^0/+^;Pyk2^−/−^ animals took significantly longer to reach the platform compared to WT mice. Data are graphed as mean ± SEM, repeated measures ANOVA with Tukey HSD multiple comparisons test, **p = 0.002, n.s. = not significant (*p* = 0.343), *n* = 9–17 mice. **C**, A 60 s probe trial was performed 24 h after the final acquisition session with the hidden platform removed. WT, Pyk2^−/−^ and PS19^0/+^ mice spent significantly greater time in the target quadrant compared to the opposite quadrant, while the difference in time spent between the target and opposite quadrants failed to reach significance for PS19^0/+^;Pyk2^−/−^ animals. Data are graphed as mean ± SEM, unpaired two-tailed *t*-test, *p < 0.05, ***p < 0.001, ****p < 0.0001, n.s. = not significant (*p* = 0.2885), *n* = 9–17 mice. Dashed line, 25%. **D**, To rule out visual impairment, latency for animals to find a platform marked with a visual cue was assessed following the probe trial. 4 animals (2 Pyk2^−/−^, 1 PS19^0/+^ and 1 PS19^0/+^;Pyk2^−/−^) were unable to locate the visible platform after 15 trials and were excluded from all MWM analyses. When excluding these animals, there were no significant differences in the time required to reach the visible platform across genotypes. Data are graphed as mean ± SEM, one-way ANOVA with Tukey’s multiple comparisons test, *n* = 9–17 mice. **E**, Animal body weights of 9–10-month-old mice across genotypes. PS19^0/+^ weighed significantly less than WT animals while PS19^0/+^;Pyk2^−/−^ animals weighed significantly less than both WT and Pyk2^−/−^ mice. Data are graphed as mean ± SEM, one-way ANOVA with Tukey’s multiple comparisons test, **p < 0.01, ****p < 0.0001, *n* = 18–23 mice. **F**, A rotarod test was performed to assess motor coordination. Latency to fall off the accelerating drum (acceleration: 0.1 rotations/min/sec; top speed: 4 rotations/min) over 5 consecutive trials was assessed and the best time (longest latency) for each animal compared. There were no significant differences in longest latency to fall across genotypes. Data are graphed as mean ± SEM, one-way ANOVA with Tukey’s multiple comparisons test, *n* = 11–17 mice. **G**, A wire hang test was conducted to assess grip strength. Animals were placed in the center of a wire grid (1 cm by 1 cm) and the latency to fall from the inverted grid was determined across 2 120 s trials. Mean latencies to fall across the 2 trials are plotted in **G.** There were no significant differences in mean latency to fall across genotypes. Data are graphed as mean ± SEM, one-way ANOVA with Tukey’s multiple comparisons test, *n* = 11–17 mice. **H**–**J**, Gait assessment was conducted using a Noldus CatWalk XT system. **H**, There were no significant differences in mean run speed across genotypes. Data are graphed as mean ± SEM, one-way ANOVA with Tukey’s multiple comparisons test, *n* = 5–11 mice. **I**, There were no significant differences in step sequence regularity index across genotypes. Data are graphed as mean ± SEM, one-way ANOVA with Tukey’s multiple comparisons test, *n* = 5–11 mice. **J**, There were no significant differences in mean maximum run variation across genotypes. Data are graphed as mean ± SEM, one-way ANOVA with Tukey’s multiple comparisons test, *n* = 5–11 mice

We sought to determine whether genetic deletion of Pyk2, found here to exacerbate Tau pathology in PS19^0/+^ mice, would result in appreciable changes in Tau-induced early-death or behavioral deficits in the same model. While, as expected, PS19^0/+^ animals demonstrated a marked reduction in survival compared to WT and Pyk2^−/−^ animals, PS19^0/+^;Pyk2^−/−^ mice showed a significant reduction in survivorship compared to PS19^0/+^ animals (Fig. 5A), and segregating survivorship data by sex suggests that this effect is primarily driven by female rather than male mice (Fig. S4). Immediately preceding death, PS19^0/+^ and PS19^0/+^;Pyk2^−/− ^animals displayed a rapid, stereotyped deterioration in condition marked by hindlimb paralysis, hunched posture and reduced body weight, all of which have been previously described in this transgenic line [48]. Immunofluorescent labeling of NeuN and pTau S202/T205 (AT8) reveals substantial Tau accumulation in the lumbar enlargement of PS19^0/+^ and PS19^0/+^;Pyk2^−/− ^spinal cord, with AT8-positive immunofluorescent signal colocalizing with NeuN-positive motor neurons in the ventral horn of PS19^0/+^ spinal cord (Fig. S5). In PS19^0/+^;Pyk2^−/−^ spinal cord, AT8 immunofluorescence is considerably more diffuse, with marked loss of NeuN-positive motor neurons in the ventral horn (Fig. S5B). The presence of Tau inclusions within motor neurons of the lumbar enlargement has been previously suggested as a causal mechanism of hindlimb paralysis in PS19^0/+^ mice, thus the reduced survivorship of PS19^0/+^;Pyk2^−/−^ compared to PS19^0/+^ mice, all of which succumbed to hindlimb paralysis, likely reflects an acceleration of PS19-mediated Tau pathology in these animals [48].

To assess the influence of Pyk2 expression on Tau-induced spatial memory impairment in PS19^0/+^ animals, we subjected 9–10-month-old animals from all four genotypes to the Morris water maze (MWM) test. While PS19^0/+^ mice of this age demonstrated no significant impairment in learning during MWM acquisition sessions, genetic deletion of Pyk2 significantly impaired MWM spatial memory acquisition in PS19^0/+^;Pyk2^−/−^ compared to WT animals (Fig. 5B). Pyk2 deletion on the WT background did not alter performance, as reported previously [11]. To assess long-term spatial memory, animals were subjected to a probe trial 24 h after the final acquisition session. WT, Pyk2^−/−^ and PS19^0/+^ animals all spent a significantly greater amount of time in the target quadrant compared to the opposite quadrant during the MWM probe trial. PS19^0/+^;Pyk2^−/−^ animals, however, failed to spend significantly more time the target quadrant compared to the opposite quadrant, suggesting that Pyk2 expression is necessary to protect against Tau-induced impairment of long-term spatial memory (Fig. 5C). Impaired spatial memory observed in PS19^0/+^;Pyk2^−/−^ mice during MWM acquisition and the probe trial could not be explained by visual impairment, as animals from all genotypes were able to effectively locate a visible platform during visual assessment (Fig. 5D). Notably, impaired spatial memory in PS19^0/+^;Pyk2^−/−^ mice occurs prior to frank hippocampal neurodegeneration in these animals, as there were no significant reductions in hippocampal cell layer thickness across genotypes (Fig. S6).

While both PS19^0/+^ and PS19^0/+^;Pyk2^−/−^ mice weighed significantly less than WT animals, Pyk2 deletion failed to result in any measurable modulation of Tau-induced body mass reduction in PS19^0/+^ mice (Fig. 5E). To assess possible Tau-induced impairments in motor coordination, grip strength and gait patterns, animals were subjected to Rotarod, wire hang and CatWalk XT assays (Fig. 5F–J). There were no observable differences across genotypes by any of these measures, suggesting that while Pyk2’s suppression of Tau pathology is concomitant with an exacerbation of Tau-induced early death and spatial memory impairment in PS19^0/+^;Pyk2^−/−^ animals, these results are not associated with any measurable sensorimotor deficits.

### Proteomic analysis reveals Pyk2’s role in regulating synaptic translational machinery, C1q expression and MAPK1 activity in PS19 mice

To more completely elucidate the effect of genetic Pyk2 deletion on PS19^0/+^ transgenic animals and to reveal the possible mechanisms by which Pyk2 suppresses Tau phosphorylation, pathology and Tau-associated phenotypes, we conducted LC-MS/MS label-free profiling of PS19^0/+^ and PS19^0/+^;Pyk2^−/−^ hippocampal tryptic peptides. Since we measured no discernable changes in either hippocampal astrogliosis or microgliosis across WT, Pyk2^−/−^, PS19^0/+^ and PS19^0/+^;Pyk2^−/−^ animals (Fig. S7), we focused our analysis on neuronal, cell-autonomous mechanisms and enhanced our analysis by utilizing hippocampal synaptosomal fractions as starting material.

**Fig. 6:**
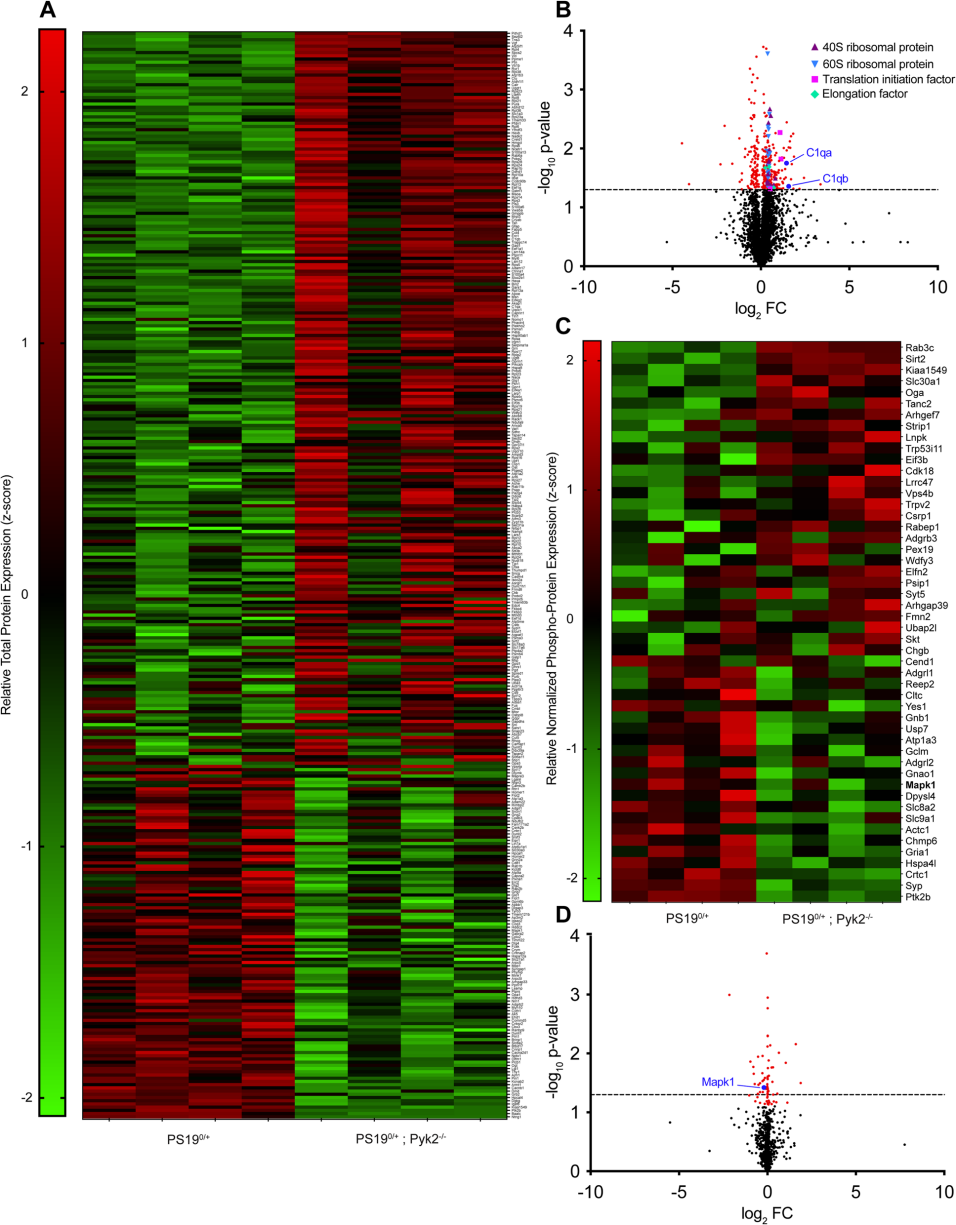
Proteomic analysis reveals signs of disrupted protein translational, increased synaptic C1q expression and decreased MAPK1 activity in PS19^0/+^;Pyk2^−/−^ animals. **A**–**D**, Synaptosomal fractions were prepared from hippocampi of 9.5–10.5-month-old PS19^0/+^ and PS19^0/+^;Pyk2^−/−^ mice and run through LC-MS/MS to identify significantly differentially regulated proteins between PS19^0/+^ and PS19^0/+^;Pyk2^−/−^ animals. **A**, Heat map showing relative abundance of significantly differentially regulated (*p* < 0.05) synaptic proteins between PS19^0/+^ and PS19^0/+^;Pyk2^−/−^ animals. **B**, Volcano plot of all total synaptic proteins identified via LC-MS/MS. Positive values for Log_2_FC represent increased synaptic protein expression in PS19^0/+^;Pyk2^−/−^ compared to PS19^0/+^ mice. Dashed line represents *p* = 0.05. Significantly differentially regulated synaptic proteins shown in red. **C**, Heat map showing relative abundance of significantly differentially regulated (*p* < 0.05), phospho-enriched, synaptic proteins (normalized to total protein abundance) between PS19^0/+^ and PS19^0/+^;Pyk2^−/−^ animals. **D**, Volcano plot of all normalized, phospho-enriched, synaptic proteins identified via LC-MS/MS. Positive values for Log_2_FC represent protein upregulation in Pyk2^−/−^ compared to WT. Dashed line represents *p* = 0.05

Proteomic analysis revealed 338 significantly differentially regulated proteins between PS19^0/+^ and PS19^0/+^;Pyk2^−/−^ hippocampal synaptosomes (Fig. 6A, B). Intriguingly, 32 (9.5%) of the identified hits were either 40S or 60S ribosomal proteins, while an additional 6 (1.8%) of identified hits were either eukaryotic translation initiation or elongation factors, all of which were upregulated in PS19^0/+^;Pyk2^−/−^ compared to PS19^0/+^ animals (Fig. 6B). Dysregulation of protein translational machinery, including altered expression of ribosomal proteins, has been observed across multiple Tau transgenic lines, and the aberrant association of Tau with ribosomal proteins, as seen in both MAPT P301L mice and AD brains, has been shown to disrupt synthesis of proteins critical for synaptic function including PSD-95 [51–54]. Notably, the expressions of both C1qa and C1qb, two components of the classical complement system, were also significantly upregulated in PS19^0/+^;Pyk2^−/−^ synaptosomes (Fig. 6B), suggesting that endogenous Pyk2 expression may protect against synaptic dysfunction through the suppression of synaptic C1q deposition.

Since Pyk2 is a protein kinase and Tau phosphorylation is correlated with pathophysiology, we specifically assessed phospho-enriched peptides obtained from PS19^0/+^ and PS19^0/+^;Pyk2^−/−^ hippocampal synaptosomes through LC-MS/MS label-free profiling. For each experimental replicate, relative abundance values for phospho-enriched hippocampal synaptosomal proteins were normalized to their respective total protein values to determine relative normalized phospho-protein abundance. We identified 50 significantly differentially regulated phospho-proteins between PS19^0/+^ and PS19^0/+^;Pyk2^−/−^ hippocampal synaptosomes (Fig. 6C, D). Of interest, the phosphorylation of MAPK1 (ERK2), a known regulator of Tau phosphorylation [35, 55–57], was significantly decreased at residue Y187 in PS19^0/+^;Pyk2^−/−^ compared to PS19^0/+^ hippocampal synaptosomes. The phosphorylation of Y187 and T185, two residues present on the activation loop of MAPK1, are required for full activation of the kinase [58, 59], and thus a decrease in MAPK1 phosphorylation at Y187 suggests a decrease in MAPK1 kinase activity in PS19^0/+^;Pyk2^−/−^ animals, which would paradoxically favor less Tau phosphorylation.

### Pyk2 expression is protective against tau-induced C1q deposition

**Fig. 7:**
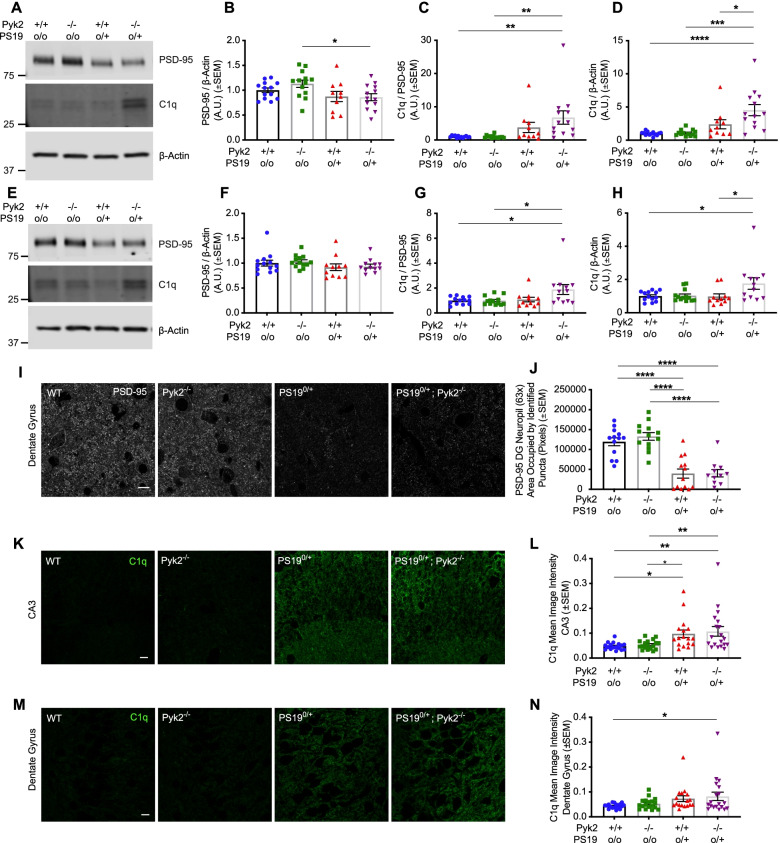
Pyk2 expression protects against Tau-mediated C1q deposition. **A**–**D**, Crude hippocampal, synaptosomal fractions were obtained from 9.5–10.5-month-old WT, Pyk2^−/−^, PS19^0/+^ and PS19^0/+^;Pyk2^−/−^ animals. Lysates were separated by SDS-PAGE and immunoblotted with the antibodies indicated. **A**, Representative immunoblot images of hippocampal, synaptosomal fractions. **B**–**D**, Quantification of **A**. A significant decrease in synaptic PSD-95 expression (normalized to β-Actin) was observed in synaptosomal fractions from PS19^0/+^;Pyk2^−/−^ hippocampi compared to those from Pyk2^−/−^ animals (**B**). When normalized to PSD-95, a significant increase in synaptic C1q expression was observed in PS19^0/+^;Pyk2^−/−^ hippocampi compared to those from WT and Pyk2^−/−^ animals (**C**). When normalized to β-Actin, an increase in synaptic C1q expression in PS19^0/+^;Pyk2^−/−^ hippocampi was significant compared to those from WT, Pyk2^−/−^ and PS19^0/+^ animals (**D**). Data are graphed as mean ± SEM, one-way ANOVA with Tukey’s multiple comparisons test, *p < 0.05, **p < 0.01, ***p < 0.001, ****p < 0.0001, *n* = 10–13 mice. **E**–**H**, Crude cortical, synaptosomal lysates were obtained from 9.5–10.5-month-old WT, Pyk2^−/−^, PS19^0/+^ and PS19^0/+^;Pyk2^−/−^ animals and immunoblots prepared as described above. **E**, Representative immunoblot images of cortical, synaptosomal fractions. **F**–**H**, Quantification of **E**. No significant changes in cortical, synaptic PSD-95 (normalized to β-Actin) were observed in synaptosomal fractions across genotypes (**F**). When normalized to β-Actin, synaptic PSD-95 expression was significantly higher in Pyk2^−/−^, PS19^0/+^ cortices compared to those from WT and Pyk2^−/−^ animals (**G**). When normalized to β-Actin, PS19^0/+^;Pyk2^−/−^ cortices demonstrated significantly higher synaptic C1q expression compared to cortices from WT and PS19^0/+^ animals (**H**). Data are graphed as mean ± SEM, one-way ANOVA with Tukey’s multiple comparisons test, *p < 0.05, *n* = 10–13 mice. **I**, Representative immunofluorescent images of PSD-95 immunoreactivity in dentate gyrus of 9.5–10.5-month-old WT, Pyk2^−/−^, PS19^0/+^ and PS19^0/+^;Pyk2^−/−^ animals. Scale bar, 10 μm. **J**, Quantification of **I**. In the dentate gyrus, both PS19^0/+^ and PS19^0/+^;Pyk2^−/−^ animals demonstrated significant reductions in the area occupied by PSD-95-positive puncta compare to WT and Pyk2^−/−^ animals (**I**). Data are graphed as mean ± SEM, one-way ANOVA with Tukey’s multiple comparisons test, ****p < 0.0001, *n* = 11–13 mice. **K**, Representative immunofluorescent images of C1q immunoreactivity in CA3 of 9.5–10.5-month-old WT, Pyk2^−/−^, PS19^0/+^ and PS19^0/+^;Pyk2^−/−^ animals. Scale bar, 10 μm. **L**, Quantification of **K**. PS19^0/+^ and PS19^0/+^;Pyk2^−/−^ animals showed significantly higher C1q immunoreactivity (mean image intensity) in the CA3 region of the hippocampus compared WT and Pyk2^−/−^ animals (**L**). Data are graphed as mean ± SEM, one-way ANOVA with Tukey’s multiple comparisons test, *p < 0.05, **p < 0.01, *n* = 17–19 mice. **M**, Representative immunofluorescent images of C1q immunoreactivity in the dentate gyrus of 9.5–10.5-month-old WT, Pyk2^−/−^, PS19^0/+^ and PS19^0/+^;Pyk2^−/−^ animals. Scale bar, 10 μm. **N**, Quantification of **M**. Only PS19^0/+^;Pyk2^−/−^ animals showed significantly higher C1q immunoreactivity in the dentate gyrus compared to WT animals. Data are graphed as mean ± SEM, one-way ANOVA with Tukey’s multiple comparisons test, *p < 0.05, *n* = 17–19 mice

From the proteomic changes, we focused our initial attention on C1q, which is a key component of the classical complement system and, in the CNS, plays a critical role in microglia-mediated synaptic pruning during development [60]. Levels of C1q are known to increase with age, and AD brains show increased neuronal deposition of C1q in both the hippocampus and frontal cortex [61]. Expression of C1q is augmented in response to both injury and Aβ exposure, and increased levels of C1q deposition at synapses are associated with microglia-mediated synaptic engulfment and synapse loss in models of neurogenerative diseases such as AD and FTD [62, 63]. We sought to biochemically validate the proteomics-based observation of increased C1qa and C1qb expression in PS19^0/+^;Pyk2^−/−^ hippocampal synaptosomes and assessed whether genetic Pyk2 deletion modulated Tau-induced C1q deposition in a manner that reflects Pyk2’s role in suppressing Tau-associated memory impairment in PS19^0/+^ mice. We obtained synaptosomal fractions from both hippocampus (Fig. 7A–D) and cortex (Fig. 7E–H) and found that PS19^0/+^;Pyk2^−/−^ mice displayed significantly higher levels of hippocampal, synaptosomal C1q (normalized to β-Actin) compared to WT, Pyk2^−/−^ and PS19^0/+^ animals. In the cortex, levels of synaptosomal C1q were significantly higher in PS19^0/+^;Pyk2^−/−^ compared to WT and PS19^0/+^ mice.

Although reduced PSD-95 expression observed in PS19^0/+^;Pyk2^−/−^ hippocampal synaptosomes only reached significance when compared to Pyk2^−/−^ mice (Fig. 7A, B), histological analysis revealed significant decreases in PSD-95-positive puncta in both PS19^0/+^ and PS19^0/+^;Pyk2^−/−^ dentate gyrus compared to WT and Pyk2^−/−^ mice (Fig. 7I, J). In the CA3 region of the hippocampus, C1q immunofluorescence signal was significantly increased in both PS19^0/+^ and PS19^0/+^;Pyk2^−/−^ animals compared to WT and Pyk2^−/−^ mice (Fig. 7K, L). However, in the dentate gyrus C1q immunofluorescence was significantly increased in PS19^0/+^;Pyk2^−/−^ animals (compared to WT), while no such increase was observed in PS19^0/+^ mice (Fig. 7M, N). Overall, the absence of Pyk2 enhances several aspects of complement-related synaptic damage in PS19 mice.

### Proteomic analysis reveals several possible regulators of tau phosphorylation modulated by Pyk2 expression

Our observations of exacerbated Tau pathology, early death and memory impairment in PS19^0/+^;Pyk2^−/−^ animals suggest that Pyk2 deletion accelerates disease progression in PS19^0/+^ transgenic mice. Identifying mechanisms by which Pyk2 may suppress Tau phosphorylation from proteomics-based comparison of aged-matched PS19^0/+^ and PS19^0/+^;Pyk2^−/−^ animals is therefore complicated by the fact that PS19^0/+^;Pyk2^−/−^ animals likely represent a more advanced stage of disease. Alterations to protein translational machinery, which likely result in substantial differences in global protein translation between PS19^0/+^ and PS19^0/+^;Pyk2^−/−^ mice, further complicate the use of these animals for the identification of Pyk2-modulated regulators of Tau phosphorylation through phospho-proteomic analysis. Considering these limitations, we also conducted LC-MS/MS label-free profiling of WT and Pyk2^−/−^ hippocampal synaptosomal peptides, as any observed changes in the synaptic proteome between animals of these genotypes would more likely reflect the influence of Pyk2 expression per se, as opposed to differences in the stage of PS19-driven pathology.

**Fig. 8:**
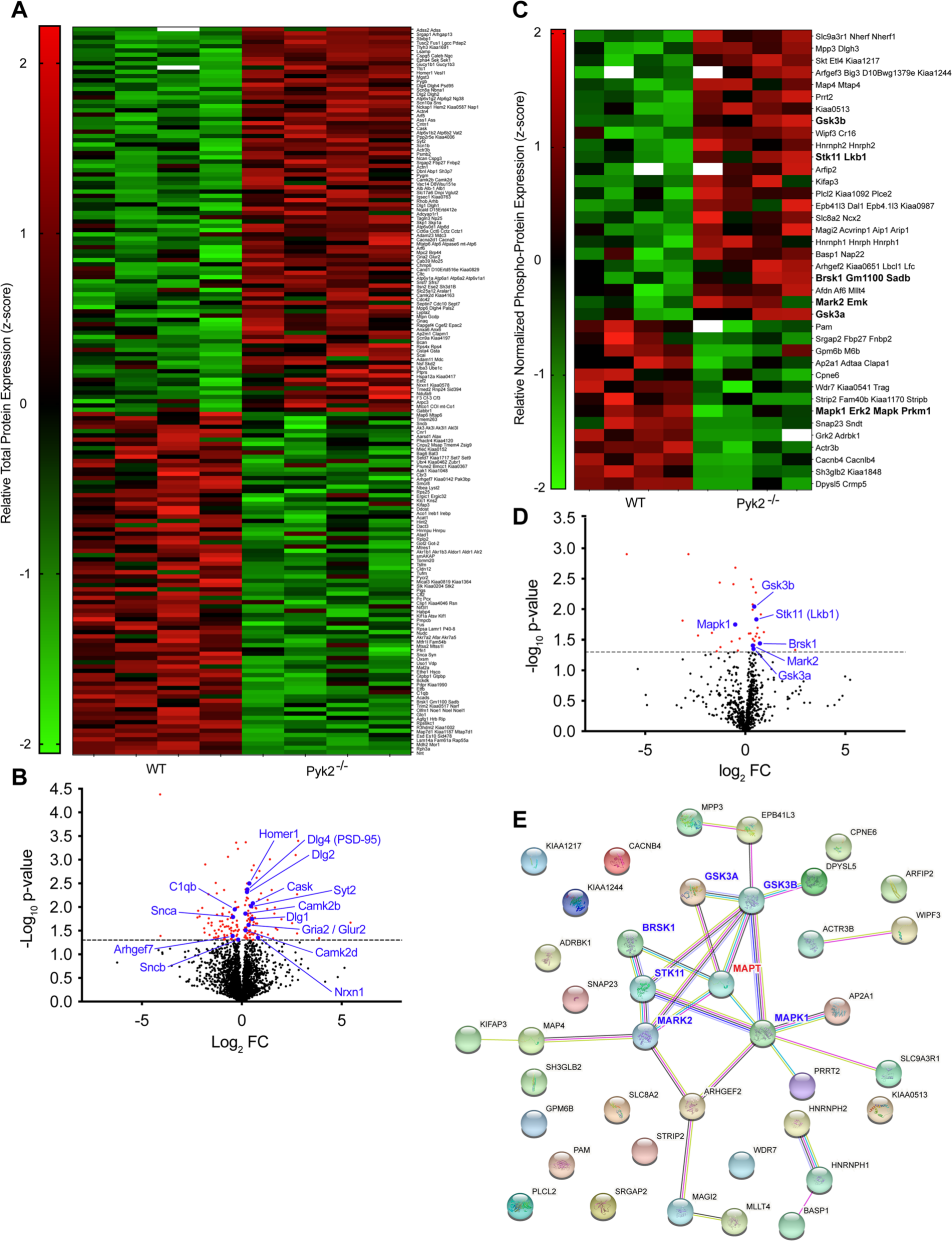
Proteomic analysis reveals potential regulators of Tau phosphorylation modulated by Pyk2. **A**–**D**, Synaptosomal fractions were prepared from hippocampi of 12-month-old WT and Pyk2^−/−^ animals and run through LC-MS/MS to identify proteins that are significantly differentially regulated by Pyk2 expression. **A**, Heat map showing relative abundance of significantly differentially regulated (*p* < 0.05) synaptic proteins between WT and Pyk2^−/−^ animals. **B**, Volcano plot of all total synaptic proteins identified via LC-MS/MS. Positive values for Log_2_FC represent protein upregulation in Pyk2^−/−^ compared to WT. Dashed line represents *p* = 0.05. Significantly differentially regulated synaptic proteins shown in red. **C**, Heat map showing relative abundance of significantly differentially regulated (*p* < 0.05), phospho-enriched, synaptic proteins (normalized to total protein abundance) between WT and Pyk2^−/−^ animals. **D**, Volcano plot of all normalized, phospho-enriched, synaptic proteins identified via LC-MS/MS. Positive values for Log_2_FC represent protein upregulation in Pyk2^−/−^ compared to WT. Dashed line represents *p* = 0.05. Significantly differentially regulated, synaptic phospho-proteins shown in red. Proximate regulators of Tau shown in blue. **E**, STRING protein-protein interaction network of all significantly differentially regulated, normalized, phospho-enriched, synaptic proteins. The interaction network was supplemented with MAPT (in red) to identify regulators of Tau. Proximate regulators of Tau (in blue) were defined as kinases or phosphatases positioned one or two degrees from MAPT. 6 kinases (and 0 phosphatases) were identified as proximate regulators of Tau modulated by Pyk2

Proteomic analysis of relative protein abundance revealed 170 significantly differentially regulated proteins between WT and Pyk2^−/−^ mice (Fig. 8A, B). Many of these hits represent potentially intriguing avenues for further exploration and may help elucidate Pyk2’s role in synaptic development and function [Dlg1, Dlg2, Dlg4 (PSD-95), Homer1, Camk2b, Camk2d, Nrxn1, Syt2, Gria2 (GluR2), Arhgef7 and Cask] and disease [Snca (α-synuclein) and Snca (β-synuclein)]. However, for the purposes of this paper, and in order explain Pyk2’s role in regulating signaling events that influence Tau phosphorylation, we focused our analysis on differential levels of phospho-peptides from the synaptosomal proteins with all values normalized to total protein abundance (Fig. 8C–E). We identified a total of 38 phospho-proteins that were significantly differentially regulated between WT and Pyk2^−/−^ animals (Fig. 8C).

In order to identify candidate regulators of Tau phosphorylation modulated by Pyk2 expression, we used STRING to generate a functional protein association network that included all 38 phospho-protein hits as well as MAPT (Tau) (Fig. 8E). Proximate regulators of Tau phosphorylation were defined a priori as kinases or phosphatases positioned one or two degrees from MAPT in the association network. Using this definition, we identified 6 possible Pyk2-modulated regulators of Tau phosphorylation (6 kinases, 0 phosphatases): Gsk3α, Gsk3β, Brsk1, Mapk1, Mark2 and Lkb1 (Stk11). Interestingly, the phosphorylation of MAPK1, the only proximate regulator of Tau phosphorylation identified in both the WT vs Pyk2^−/−^ and the PS19^0/+^ vs PS19^0/+^;Pyk2^−/−^ phospho-proteomic analyses, was also decreased in Pyk2^−/−^ hippocampal synaptosomes. However, since the activity of this kinase was decreased (rather than increased) with Pyk2 deletion, it is unlikely that this putative Tau kinase would explain Pyk2’s ability to suppress Tau phosphorylation.

### GO enrichment analysis reveals Pyk2-dependent modulation of multiple biological pathways unique to PS19 animals

**Fig. 9:**
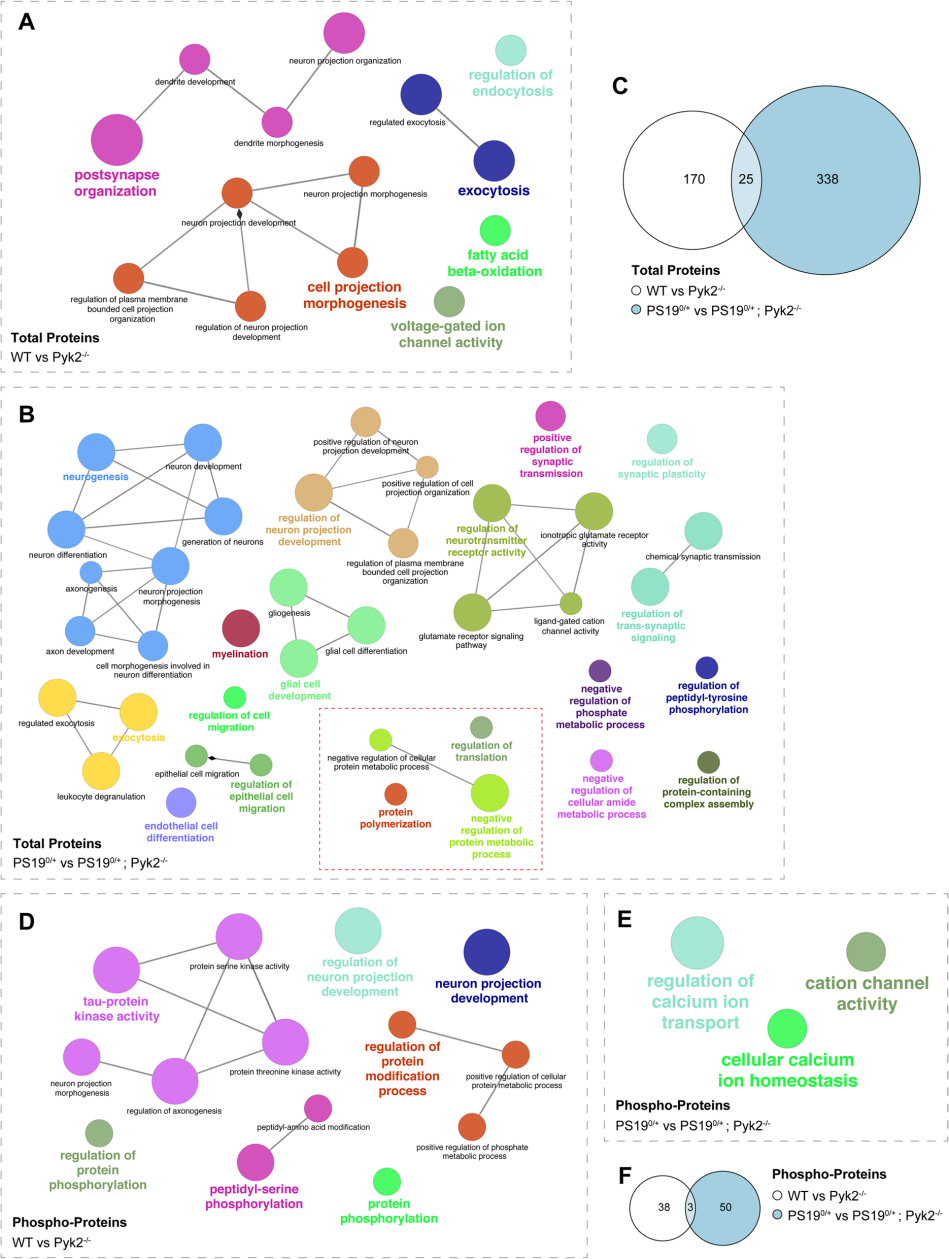
Pathway enrichment of proteins with Pyk2-regulated expression. **A**–**B**, Functional networks of identified proteomic hits (total protein fraction) from WT vs Pyk2^−/−^ (**A**) and PS19^0/+^ vs PS19^0/+^;Pyk2^−/−^ (**B**) analyses generated in ClueGO. Pathways involved in protein translation are boxed in red. **C**, Venn Diagram showing overlap of significant total protein hits between analyses. **D**–**E**, Functional networks of phospho-proteomic hits (normalized phospho-enriched fraction) from WT vs Pyk2^−/−^ (**D**) and PS19^0/+^ vs PS19^0/+^;Pyk2^−/−^ (**E**) analyses. **F**, Venn Diagram showing overlap of significant normalized phospho-enriched protein hits between analyses

To further understand the pattern of differential protein abundance and phosphorylation in hippocampal synaptosomes, we conducted Gene Ontology (GO) enrichment analysis and generated functionally-grouped network maps of significantly differentially regulated synaptic proteins identified across each proteomic and phospho-proteomic analysis. Network maps showing clusters of significantly enriched pathways (*p* < 0.05) were generated in ClueGO using the GO Biological Process ontology with network parameters for each analysis kept constant for proteomic (total protein) and phospho-proteomic (normalized phospho-enriched protein) hits, respectively (Fig. 9A, B, D, E). Across proteomic analyses of total hippocampal synaptosomal proteins, 25 proteins were shared between WT vs Pyk2^−/−^ and PS19^0/+^ vs PS19^0/+^;Pyk2^−/−^ animals, representing 14.7% of hits identified in the WT vs Pyk2^−/−^ analysis (Fig. 9C). GO enrichment of total proteins reveals several biological pathways conserved between WT vs Pyk2^−/−^ and PS19^0/+^ vs PS19^0/+^;Pyk2^−/−^ proteomic analyses, reflecting Pyk2’s role in regulating neuronal projection (GO terms: regulation of neuron projection development; regulation of plasma membrane bounded cell projection organization; regulation of plasma membrane bounded cell projection organization) and exocytosis (GO terms: regulated exocytosis; exocytosis) (Fig. 9A, B, Table 1).

**Table 1:** Identified Gene Ontology (GO) enrichment pathways of total protein hits across proteomic analyses. Identified pathways (nodes) are sorted by analysis (WT vs Pyk2^−/−^ and PS19^0/+^ vs PS19^0/+^;Pyk2^−/−^) and by percent associated genes. Common pathways between analyses are bolded

Fraction	Analysis	% Associated Genes	Associated Genes Found	Number of Genes	Pathway Term	GO ID	Term ***P***-Value	Term ***P***-Value Corrected with Bonferroni Step Down
Total Protein	WT vs Pyk2^−/−^	6.10	ABCD2, ACADS, ACAT1, CNR1, ETFB	5	fatty acid beta-oxidation	GO:0006635	7.55E-05	0.007321993
		6.06	ABCD2, CDC42, DBNL, DLG4, EPHA4, KIF1A	6	neuron projection organization	GO:0106027	1.45E-05	0.001450712
		4.92	ACTN1, ARF6, CDC42, DBNL, DLG1, DLG4, EPHA4, KIF1A, PTPRS	9	postsynapse organization	GO:0099173	5.63E-07	5.68E-05
		3.90	CDC42, DBNL, DLG4, EPHA4, KIF1A, MAP 6	6	dendrite morphogenesis	GO:0048813	1.72E-04	0.015991856
		3.10	AAK1, AP2M1, ARF6, ATAD1, CDC42, DLG4, TF	7	regulation of endocytosis	GO:0030100	2.03E-04	0.018696253
		3.02	ARF6, CDC42, DBNL, DLG4, EPHA4, KIF1A, MAP 6, PTPRS	8	dendrite development	GO:0016358	8.42E-05	0.008083191
		2.75	CNR1, DLG1, DLG2, DLG4, GRIA2, SCN1B, SCN8A, SCN9A	8	voltage-gated ion channel activity	GO:0005244	1.61E-04	0.015097501
		2.12	ARF6, BRSK1, CNR1, CNTN1, DBNL, EPHA4, KIF1A, MAP 6, PTPRS, SCN1B	10	**regulation of neuron projection development**	GO:0010975	2.05E-04	0.018639542
		1.79	BRSK1, CDC42, CSPG5, DBNL, DLG4, EPHA4, ITSN2, KIF1A, MAP 6, OLFM1, PTPRS, SCN1B, SYT2	13	cell projection morphogenesis	GO:0048858	1.22E-04	0.011542529
		1.73	ACTN1, ACTN4, CAB39, CAND1, CNR1, CSPG5, DBNL, DDOST, EEF2, HABP4, MLEC, PYGB, SYT2, TF, UBR4	15	**regulated exocytosis**	GO:0045055	4.99E-05	0.004892033
		1.73	ACTN1, ACTN4, CAB39, CAND1, CHMP6, CNR1, CSPG5, DBNL, DDOST, EEF2, HABP4, MLEC, NSF, PYGB, SYT2, TF, UBR4	17	**exocytosis**	GO:0006887	1.48E-05	0.001465131
		1.72	ARF6, BRSK1, CDC42, CNR1, CNTN1, DBNL, EPHA4, KIF1A, MAP 6, PTPRS, SCN1B, SEPTIN7	12	**regulation of plasma membrane bounded cell projection organization**	GO:0120035	3.24E-04	0.029116126
		1.69	BRSK1, CDC42, DBNL, DLG4, EPHA4, ITSN2, KIF1A, MAP 6, OLFM1, PTPRS, SCN1B, SYT2	12	**neuron projection morphogenesis**	GO:0048812	3.68E-04	0.032738513
		1.40	ARF6, BRSK1, CDC42, CNR1, CNTN1, DBNL, DLG4, EPHA4, ITSN2, KIF1A, MAP 6, OLFM1, PTPRS, SCN1B, SYT2	15	neuron projection development	GO:0031175	5.08E-04	0.044721907
	PS19^0/+^ vs PS19^0/+^;Pyk2^−/−^	10.34	DLG2, DLG4, MINK1, NLGN3, NPTX1, OPRM1, PRRT1, PTK2B, SRC	9	regulation of neurotransmitter receptor activity	GO:0099601	8.49E-08	2.16E-05
		9.88	DLG2, DLG4, GRIK4, MINK1, NLGN3, OPRM1, PRRT1, PTK2B	8	ionotropic glutamate receptor activity	GO:0004970	6.51E-07	1.62E-04
		8.26	ABCA2, C1QA, CD9, DMD, GFAP, GRN, GSTP1, PRDX6, SIRT2, TSPAN2	10	glial cell development	GO:0021782	1.38E-07	3.48E-05
		7.76	DLG2, DLG4, GRIK4, MINK1, NLGN3, OPRM1, PLCB1, PRRT1, PTK2B	9	glutamate receptor signaling pathway	GO:0007215	1.01E-06	2.51E-04
		7.09	ABCA2, CD9, HEXB, MTOR, PRDX6, PTN, SCN2A, SIRT2, TSPAN2, UGT8	10	myelination	GO:0042552	5.76E-07	1.44E-04
		6.67	DMD, PLCB1, PTN, PTPRS, RAB1B, RAP1B, RAP2B, TJP2	8	endothelial cell differentiation	GO:0045446	1.27E-05	0.003012572
		5.88	APOE, DLG4, FLOT1, GFAP, LGI1, NLGN3, PRRT1, PTK2B, PTN, SYT12	10	positive regulation of synaptic transmission	GO:0050806	3.17E-06	7.63E-04
		5.75	APBB1, APOE, CNTN1, DMD, EHD1, GRN, MTOR, OPA1, PTK2B, PTN	10	positive regulation of neuron projection development	GO:0010976	3.90E-06	9.36E-04
		5.34	APOE, CPEB3, CPLX2, DLG4, ERC2, GFAP, PRRT1, PTK2B, PTN, SYT12, VGF	11	regulation of synaptic plasticity	GO:0048167	2.59E-06	6.30E-04
		5.06	ABCA2, C1QA, CD9, DMD, GFAP, GRN, GSTP1, MTOR, PRDX6, PTN, SIRT2, TSPAN2	12	glial cell differentiation	GO:0010001	1.57E-06	3.84E-04
		5.02	APOE, CPEB3, CPLX2, DLG4, ERC2, FABP5, FLOT1, GFAP, GRIK4, LGI1, MTOR, NLGN3, NPTX1, NTNG1, PLCB1, PRRT1, PTK2B, PTN, PTPRS, RAP1B, SRC, SYT12, VGF	23	regulation of trans-synaptic signaling	GO:0099177	2.19E-11	5.74E-09
		4.63	DLG2, DLG4, DMD, GRIK4, MINK1, NLGN3, OPRM1, PRRT1, PTK2B, RYR1	10	ligand-gated cation channel activity	GO:0099094	2.59E-05	0.006110782
		4.48	APOE, CALR, CPEB3, EDC4, LARP1, PIN1, PURA, RACK1, RTN1, UPF1	10	negative regulation of cellular amide metabolic process	GO:0034249	3.40E-05	0.007919981
		4.42	ABCA2, C1QA, CD9, DMD, GFAP, GRN, GSTP1, HEXB, MTOR, PRDX6, PTK2B, PTN, SIRT2, TSPAN2	14	gliogenesis	GO:0042063	1.06E-06	2.62E-04
		4.20	APOE, CALR, CSNK2B, GRN, MTOR, PLPP3, PPM1F, PTK2B, PTN, SRC	10	regulation of epithelial cell migration	GO:0010632	5.89E-05	0.013542207
		3.60	APBB1, APOE, CNTN1, DGKG, DMD, EHD1, FKBP4, GFAP, GRN, MTOR, NTNG1, OPA1, PKN1, PTK2B, PTN, PTPRS, THY1	17	**regulation of neuron projection development**	GO:0010975	1.23E-06	3.02E-04
		3.59	APOE, CPEB3, CPLX2, DLG2, DLG4, ERC2, FLOT1, GFAP, GRIK4, LGI1, LIN7A, MINK1, MTOR, NLGN3, NPTX1, NTNG1, OPRM1, PLCB1, PRRT1, PTK2B, PTN, PTPRS, RAP1B, SLC18A3, SRC, SYPL1, SYT12, VGF	28	chemical synaptic transmission	GO:0007268	3.09E-10	8.07E-08
		3.58	CADM4, CD81, CNTN1, DLG4, MTOR, PTK2B, RACK1, RAP2B, SRC, THY1	10	regulation of peptidyl-tyrosine phosphorylation	GO:0050730	2.17E-04	0.048624534
		3.54	APOE, CALR, CSNK2B, GRN, MTOR, PKN1, PLPP3, PPM1F, PTK2B, PTN, SRC	11	epithelial cell migration	GO:0010631	1.18E-04	0.026898848
		3.45	APBB1, APOE, CNTN1, DLG4, DMD, EHD1, GRN, MTOR, OPA1, PKN1, PTK2B, PTN, SRC	13	positive regulation of cell projection organization	GO:0031346	3.66E-05	0.008487912
		3.37	ARL6, ARPC5, CLIP1, FKBP4, GRB2, MTOR, OPA1, PIN1, PKN1, PTK2B, TPPP3	11	protein polymerization	GO:0051258	1.78E-04	0.040236304
		3.22	APBB1, APOE, FLOT1, GRB2, GRN, LGI1, NLGN3, NPTX1, NTNG1, OLFM1, PKN1, PTN, PTPRS, RHOG, S100A6, SRC, THY1, TSPAN2	18	axon development	GO:0061564	2.88E-06	6.96E-04
		3.19	AMPD3, ARPC5, BIN2, CPLX2, CSNK2B, CTSA, DDOST, FABP5, GRN, GSTP1, HEXB, PA2G4, PRDX6, PTGES2, RAB6A, RAP1B, RAP2B, RHOG, VAT1	19	leukocyte degranulation	GO:0043299	1.73E-06	4.23E-04
		3.16	AMPD3, ANXA5, ARPC5, ATP9A, BIN2, CD9, CPLX2, CSNK2B, CTSA, DDOST, ERC2, FABP5, GRN, GSTP1, HABP4, HEXB, LIN7A, PA2G4, PKN1, PRDX6, PTGES2, RAB6A, RAP1B, RAP2B, RHOG, SYT12, SYT17, TLN1, VAT1, VPS4A, VTI1B	31	**exocytosis**	GO:0006887	7.34E-10	1.90E-07
		3.12	APOE, ARPC5, CLIP1, CRYAB, FKBP4, GFAP, GRB2, IRGM, MTOR, PKN1, PTK2B, RACK1, RAP1B, SRC, TPPP3	15	regulation of protein-containing complex assembly	GO:0043254	2.93E-05	0.006858316
		3.12	AMPD3, ANXA5, ARPC5, BIN2, CD9, CPLX2, CSNK2B, CTSA, DDOST, ERC2, FABP5, GRN, GSTP1, HABP4, HEXB, PA2G4, PRDX6, PTGES2, RAB6A, RAP1B, RAP2B, RHOG, SYT12, SYT17, TLN1, VAT1, VTI1B	27	**regulated exocytosis**	GO:0045055	1.41E-08	3.61E-06
		3.11	APBB1, APOE, DLG4, DMD, FLOT1, GRB2, LGI1, MINK1, NLGN3, NPTX1, NTNG1, OLFM1, OPA1, PKN1, PTN, PTPRS, RHOG, S100A6, SRC, SYT17, THY1, UGT8	22	**neuron projection morphogenesis**	GO:0048812	3.78E-07	9.50E-05
		2.97	APBB1, APOE, DLG4, FLOT1, GRB2, LGI1, MINK1, NLGN3, NPTX1, NTNG1, OLFM1, OPA1, PKN1, PTN, PTPRS, RHOG, S100A6, SRC, THY1	19	cell morphogenesis involved in neuron differentiation	GO:0048667	4.88E-06	0.001166078
		2.95	CALR, CPEB3, EDC4, EEF1D, EEF1G, EIF3B, HABP4, LARP1, MTOR, PA2G4, PTK2B, PURA, RACK1, UPF1	14	regulation of translation	GO:0006417	9.95E-05	0.022778961
		2.94	APBB1, APOE, FLOT1, GRB2, LGI1, NLGN3, NPTX1, NTNG1, OLFM1, PKN1, PTPRS, RHOG, S100A6, SRC, THY1	15	axonogenesis	GO:0007409	5.71E-05	0.013191499
		2.79	APBB1, APOE, C1QA, CNTN1, CPEB3, DGKG, DLG4, DMD, EHD1, FKBP4, FLOT1, GFAP, GPM6B, GRB2, GRN, LGI1, MINK1, MTOR, NLGN3, NPTX1, NTNG1, OLFM1, OPA1, PKN1, PTK2B, PTN, PTPRS, RHOG, S100A6, SRC, SYT17, THY1, TSPAN2, UGT8	34	neuron development	GO:0048666	2.33E-09	6.02E-07
		2.78	ABCA2, APOE, CADM4, DMD, GSTP1, MTOR, PKN1, PLPP3, PPM1F, PPME1, PTN, RACK1, SIRT2, THY1	14	negative regulation of phosphate metabolic process	GO:0045936	1.84E-04	0.041490973
		2.72	APBB1, APOE, CNTN1, DGKG, DMD, EHD1, FKBP4, GFAP, GRN, MTOR, NTNG1, OPA1, PKN1, PTK2B, PTN, PTPRS, RHOG, SRC, THY1	19	**regulation of plasma membrane bounded cell projection organization**	GO:0120035	1.66E-05	0.003934112
		2.58	APCS, APOE, CADM4, CALR, CNTN1, CPEB3, CRYAB, CTSA, DMD, EDC4, ERC2, FLOT2, GSTP1, LARP1, MTOR, OGT, PFDN1, PIN1, PKN1, PLPP3, PPM1F, PPME1, PTN, PURA, RACK1, RTN1, SIRT2, SRC, THY1, UPF1	30	negative regulation of protein metabolic process	GO:0051248	1.41E-07	3.55E-05
		2.46	ABCA2, APBB1, APOE, C1QA, CALR, CD9, CNTN1, CPEB3, DGKG, DLG4, DMD, EHD1, FKBP4, FLOT1, GFAP, GPM6B, GRB2, GRN, GSTP1, HEXB, LGI1, MINK1, MTOR, NLGN3, NPTX1, NTNG1, OLFM1, OPA1, OPRM1, PIN1, PKN1, PRDX6, PTK2B, PTN, PTPRS, RHOG, S100A6, SIRT2, SRC, SYT17, THY1, TSPAN2, UGT8	43	neurogenesis	GO:0022008	5.77E-10	1.50E-07
		2.43	APBB1, APOE, C1QA, CALR, CNTN1, CPEB3, DGKG, DLG4, DMD, EHD1, FKBP4, FLOT1, GFAP, GPM6B, GRB2, GRN, LGI1, MINK1, MTOR, NLGN3, NPTX1, NTNG1, OLFM1, OPA1, PIN1, PKN1, PTK2B, PTN, PTPRS, RHOG, S100A6, SRC, SYT17, THY1, TSPAN2, UGT8	36	neuron differentiation	GO:0030182	2.77E-08	7.09E-06
		2.37	APOE, CALR, CD81, CD9, CNTN1, CSNK2B, GRN, GSTP1, MINK1, MTOR, NTNG1, PKN1, PLCB1, PLPP3, PPM1F, PTK2B, PTN, RACK1, RAP2B, RHOG, SRC, THY1	22	regulation of cell migration	GO:0030334	2.87E-05	0.006756045
		2.33	APBB1, APOE, C1QA, CALR, CNTN1, CPEB3, DGKG, DLG4, DMD, EHD1, FKBP4, FLOT1, GFAP, GPM6B, GRB2, GRN, LGI1, MINK1, MTOR, NLGN3, NPTX1, NTNG1, OLFM1, OPA1, OPRM1, PIN1, PKN1, PTK2B, PTN, PTPRS, RHOG, S100A6, SIRT2, SRC, SYT17, THY1, TSPAN2, UGT8	38	generation of neurons	GO:0048699	3.20E-08	8.16E-06
		2.11	APOE, CADM4, CALR, CPEB3, CRYAB, DMD, EDC4, ERC2, GSTP1, LARP1, MTOR, OGT, PKN1, PLPP3, PPM1F, PPME1, PTN, PURA, RACK1, SIRT2, SRC, THY1, UPF1	23	negative regulation of cellular protein metabolic process	GO:0032269	1.53E-04	0.034655683

Several additional pathways, however, were unique to the PS19^0/+^ vs PS19^0/+^;Pyk2^−/−^ analysis, likely reflecting Pyk2’s role in regulating aberrant, Tau-associated cellular processes specific to PS19^0/+^ mice, including pathways involved in protein translation and processing (GO terms: regulation of translation; protein polymerization; negative regulation of protein metabolic process; negative regulation of cellular protein metabolic process) (Fig. 9B). Also unique to the PS19^0/+^ vs PS19^0/+^;Pyk2^−/−^ analysis are pathways involved in glial development (GO terms: glial cell development, glial cell differentiation; gliogenesis), myelination (GO term: myelination) and synaptic homeostasis (GO terms: positive regulation of synaptic transmission; regulation of synaptic plasticity; regulation of trans-synaptic signaling; chemical synaptic transmission), the latter of which may reflect impaired synaptic function in PS19^0/+^;Pyk2^−/−^ animals. Using Kyoto Encyclopedia of Genes and Genomes (KEGG) enrichment ontology for total protein hits identifies a single node for the PS19^0/+^ vs PS19^0/+^;Pyk2^−/−^ proteomic analysis (GO term: pathways of neurodegeneration), while no pathways were identified for the WT vs Pyk2^−/−^ analysis using the same parameters, suggesting that Pyk2 deletion alters pathways related to neurodegeneration in PS19^0/+^, but not WT animals (Table S1).

We also generated functionally-grouped network maps for normalized phospho-enriched protein hits across phospho-proteomic analyses (Fig. 9 D, E). Encouragingly, GO enrichment analysis of WT vs Pyk2^−/−^ hits revealed significant enrichment for multiple biological pathways involved in protein phosphorylation (GO terms: tau-protein kinase activity; protein serine kinase activity; protein threonine kinase activity; regulation of protein phosphorylation; peptidyl-serine phosphorylation; protein phosphorylation), with “tau-protein kinase activity” having the highest percentage of associated genes of any identified pathway (11.43%) (Table 2). Using the same network parameters, we found considerably fewer enriched pathways from hits identified from the PS19^0/+^ vs PS19^0/+^;Pyk2^−/−^ analysis, with all significantly enriched pathways relating to cation homeostasis (GO terms: regulation of calcium ion transport; cellular calcium ion homeostasis; cation channel activity), likely reflecting Pyk2’s role in regulating calcium homeostasis in PS19^0/+^ transgenic mice (Fig. 9E). Only 3 hits (7.9% of WT vs Pyk2^−/−^ hits) were shared between phospho-proteomic analyses (Fig. 9F). The lack of enriched pathways relating to protein phosphorylation in the PS19^0/+^ vs PS19^0/+^;Pyk2^−/−^ analysis further suggests that phospho-proteomic hits that may mechanistically explain Pyk2’s role in suppressing Tau phosphorylation are likely masked in PS19^0/+^ animals as pathology progresses.

**Table 2:** Identified Gene Ontology (GO) enrichment pathways of normalized phospho-enriched protein hits across phospho-proteomic analyses. Identified pathways (nodes) are sorted by analysis (WT vs Pyk2^−/−^ and PS19^0/+^ vs PS19^0/+^;Pyk2^−/−^) and by percent associated genes

Fraction	Analysis	% Associated Genes	Associated Genes Found	Number of Genes	Pathway Term	GO ID	Term ***P***-Value	Term ***P***-Value Corrected with Bonferroni Step Down
Normalized Phospho-Enriched Protein	WT vs Pyk2^−/−^	11.43	BRSK1, GSK3A, GSK3B, MARK2	4	tau-protein kinase activity	GO:0050321	4.49E-08	5.39E-07
		2.33	BRSK1, GSK3B, MARK2, STK11	4	regulation of axonogenesis	GO:0050770	2.76E-05	2.76E-04
		1.48	AP2A1, BRSK1, GSK3A, GSK3B, MAGI2, MARK2, STK11	7	regulation of neuron projection development	GO:0010975	3.06E-07	3.37E-06
		1.31	BRSK1, GSK3A, GSK3B, MARK2, STK11	5	protein serine kinase activity	GO:0106310	3.71E-05	3.34E-04
		1.31	BRSK1, GSK3A, GSK3B, MARK2, STK11	5	protein threonine kinase activity	GO:0106311	3.71E-05	3.34E-04
		1.19	GSK3A, GSK3B, MARK2, PLCL2	4	peptidyl-serine phosphorylation	GO:0018105	3.62E-04	0.002893702
		0.93	AP2A1, BRSK1, CPNE6, GPM6B, GSK3A, GSK3B, MAGI2, MAP 4, MARK2, STK11	10	neuron projection development	GO:0031175	2.92E-08	3.79E-07
		0.71	BRSK1, CPNE6, GSK3B, MARK2, STK11	5	neuron projection morphogenesis	GO:0048812	6.72E-04	0.004700896
		0.47	GSK3A, MAGI2, MARK2, MPP3, STK11	5	positive regulation of phosphate metabolic process	GO:0045937	0.004069528	0.012208583
		0.41	BRSK1, GSK3A, GSK3B, MARK2, MPP3, PLCL2, STK11	7	protein phosphorylation	GO:0006468	0.001289881	0.007739284
		0.41	GSK3A, GSK3B, MAGI2, MARK2, MPP3, PLCL2, STK11	7	regulation of protein modification process	GO:0031399	0.001294324	0.006471619
		0.39	GSK3A, MARK2, MPP3, PLCL2, STK11	5	regulation of protein phosphorylation	GO:0001932	0.008870538	0.017741077
		0.39	GSK3A, GSK3B, MAGI2, MARK2, MPP3, STK11	6	positive regulation of cellular protein metabolic process	GO:0032270	0.00389758	0.015590322
		0.36	GSK3A, GSK3B, MARK2, MPP3, PLCL2	5	peptidyl-amino acid modification	GO:0018193	0.011955289	0.011955289
	PS19^0/+^ vs PS19^0/+^;Pyk2^−/−^	1.54	GNAO1, PTK2B, SLC30A1, TRPV2	4	regulation of calcium ion transport	GO:0051924	4.89E-04	0.003424011
		0.89	GRIA1, PTK2B, SCN2A, SLC30A1, TRPV2	5	cation channel activity	GO:0005261	0.001096794	0.006580766
		0.85	GRIA1, PTK2B, SLC30A1, TRPV2	4	cellular calcium ion homeostasis	GO:0006874	0.004364709	0.021823543

### Pyk2 inhibits LKB1 (SKT11) and p38 MAPK activity

Because LKB1 is implicated in both Aβ processing as well as Tau phosphorylation, and thus, like Pyk2, is well-positioned to serve as possible mechanistic link between Aβ and Tau, we selected this kinase for further validation [64–66]. To confirm whether Pyk2 modulates LKB1 (STK11) activity, we inhibited Pyk2 pharmacologically in mature iPSC-derived human cortical neurons and measured the phosphorylation of LKB1 biochemically at S428. The phosphorylation of this serine residue, located within the C-terminal domain of LKB1, is required for LKB1 nuclear export, substrate biding and activation [67–69]. Since evidence of LKB1’s ability to directly phosphorylate Tau is lacking, we also measured the activity of p38 MAPK, a known downstream effector of LKB1 signaling [32, 33] and a direct kinase of Tau [34–36], through the phosphorylation of T180/Y182. The phosphorylation of p38 MAPK at T180/Y182, two auto-phosphorylated residues present in the protein’s activation loop required for catalytic activity and substrate binding, are well-validated markers of p38 MAPK activity [70–73].

**Fig. 10:**
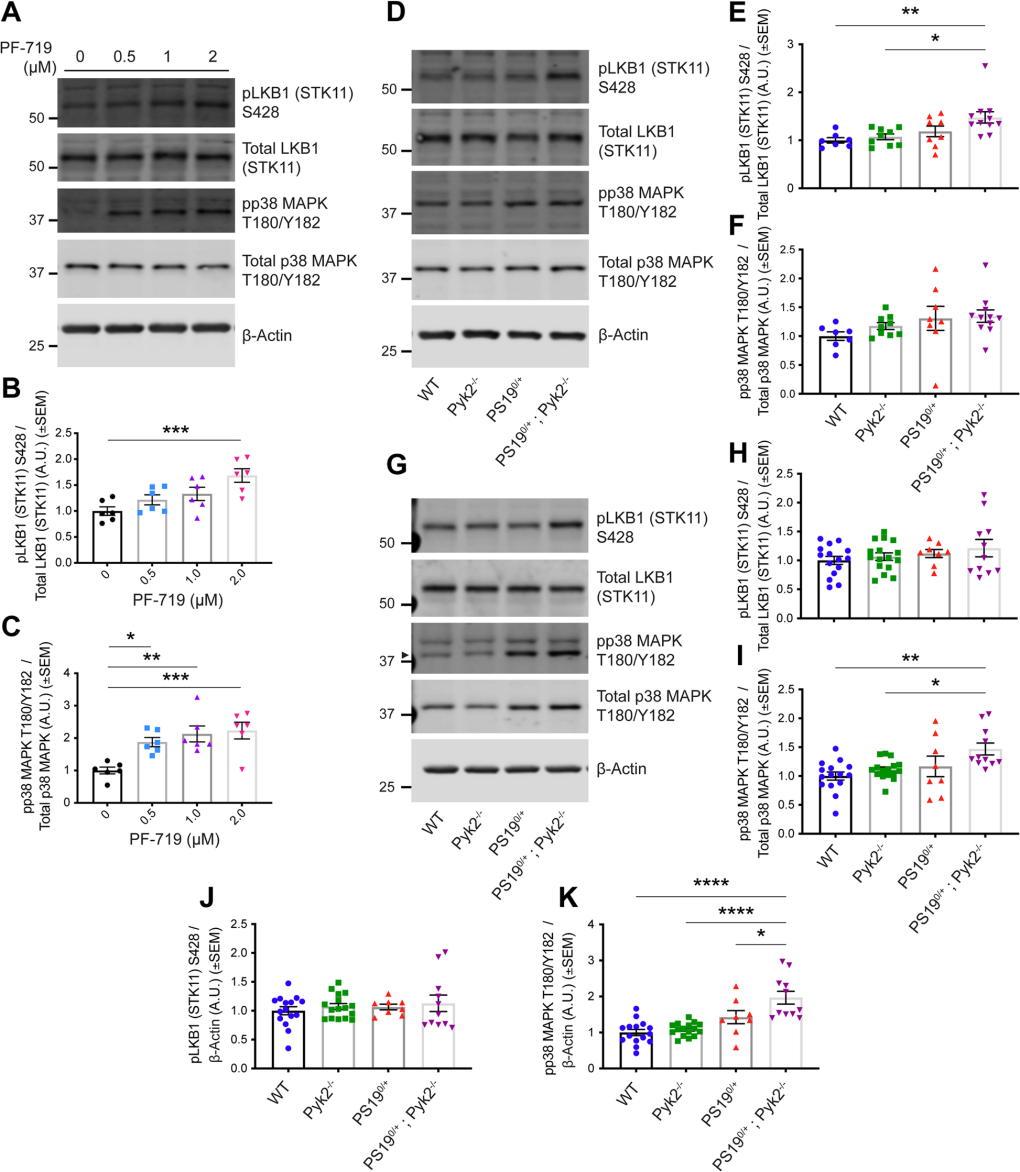
Pyk2 inhibits LKB1 and p38 MAPK activity. **A**–**C**, iPSC-derived human cortical neurons (90–100 days post terminal differentiation) (same as shown in Fig. 2E–I) were treated with PF-719 at indicated concentrations for 2 h at 37 °C and, immediately following treatment, homogenized in RIPA containing 1% SDS. Lysates were separated by SDS-PAGE and immunoblotted with the LKB1 and p38 MAPK antibodies indicated. **A**, Representative immunoblot images of lysates from PF-719-treated iPSC-derived human cortical neurons. **B** and **C**, Quantification of **A**. Pyk2 inhibition significantly increased LKB1 activity (pLKB1 S428 normalized to total LKB1) at 2.0 μM PF-719 (**B**) and significantly increased p38 MAPK activity (pp38 MAPK T180/Y182 normalized to total p38 MAPK) at every concentration of PF-719 (**C**). Data are graphed as mean ± SEM, one-way ANOVA with Dunnett’s multiple comparisons test, *p < 0.05, **p < 0.01, ***p < 0.001, *n* = 6. **D**–**F**, TBS-soluble lysates from hippocampi of 9.5–10.5-month-old WT, Pyk2^−/−^, PS19^0/+^ and PS19^0/+^;Pyk2^−/−^ animals were separated by SDS-PAGE and immunoblotted with the LKB1 and p38 MAPK antibodies listed. **D**, Representative immunoblot images of TBS-soluble hippocampal lysates. **E** and **F**, Quantification of **D**. A significant increase in TBS-soluble LKB1 activity (pLKB1 S428 normalized to total LKB1) is observed in PS19^0/+^;Pyk2^−/−^ animals compared to WT and Pyk2^−/−^ animals (**E**), while there were no significant differences in TBS-soluble MAPK activity (pp38 MAPK T180/Y182 normalized to total p38 MAPK) observed across genotypes (**F**). Data are graphed as mean ± SEM, one-way ANOVA with Tukey’s multiple comparisons test, *p < 0.05, **p < 0.01, *n* = 7–11 mice. **G**–**K**, TBS-insoluble, SDS-soluble lysates from hippocampi of 9.5–10.5-month-old WT, Pyk2^−/−^, PS19^0/+^ and PS19^0/+^;Pyk2^−/−^ animals were separated by SDS-PAGE and immunoblotted with the antibodies indicated. **G**, Representative immunoblot images of TBS-insoluble, SDS-soluble hippocampal lysates. Arrowhead indicates pp38 MAPK T180/Y182. **H**–**K**, Quantification of **G**. While no significant differences in TBS-insoluble, SDS-soluble LKB1 activity (pLKB1 S428 normalized to total LKB1) were observed across genotypes (**H**), PS19^0/+^;Pyk2^−/−^ animals demonstrated a significant increase in TBS-insoluble, SDS-soluble p38 MAPK activity (pp38 MAPK T180/Y182 normalized to total p38 MAPK) compared to WT and Pyk2^−/−^ animals (**I**). No differences were observed in absolute levels of TBS-insoluble, SDS-soluble phospho-LKB1 (pLKB1 S428 normalized to β-Actin) across genotypes (**J**), however PS19^0/+^;Pyk2^−/−^ exhibited a significant increase in absolute levels of TBS-insoluble, SDS-soluble phosho-p38 MAPK (pp38 MAPK T180/Y182 normalized to β-Actin) compared to WT, Pyk2^−/−^ and PS19^0/+^ animals (**K**). Data are graphed as mean ± SEM, one-way ANOVA with Tukey’s multiple comparisons test, *p < 0.05, **p < 0.01, ****p < 0.0001, *n* = 8–16 mice

In iPSC-derived human cortical neurons, Pyk2 inhibition with PF-719 significantly increased both the phosphorylation of LKB1 at S428 as well as the phosphorylation of p38 MAPK at T180/Y182, suggesting that basal levels of neuronal Pyk2 activity suppress the activation of both LKB1 and p38 MAPK in a manner that reflects Py2k’s ability to inhibit Tau phosphorylation (Fig. 10A–C). Since the phosphorylation of MAPK1 was significantly differentially regulated in both WT vs Pyk2^−/−^ and PS19^0/+^ vs PS19^0/+^;Pyk2^−/−^ phospho-proteomic analyses, we also assessed whether pharmacological Pyk2 inhibition would modulate MAPK1 activity via phosphorylation of its activation loop at T185/Y187 in iPSC-derived human cortical neurons. Consistent with our phospho-proteomic results suggesting decreased phosphorylation of MAPK1 in both Pyk2 and PS19^0/+^;Pyk2^−/−^ hippocampal synaptosomes, we observed significant inhibition of MAPK1 phosphorylation at T185/Y187 with pharmacological inhibition of Pyk2, suggesting that Pyk2 positively regulates the activity of MAPK1 (Fig. S8).

We also assessed the activation of LKB1 and p38 MAPK in hippocampal tissue from 9.5–10-month old WT, Pyk2^−/−^, PS19^0/+^ and PS19^0/+^;Pyk2^−/−^ animals (Fig. 10D–K). TBS-soluble LKB1 demonstrated significantly higher activation (pLKB1 S428 normalized to total LKB1) in PS19^0/+^;Pyk2^−/−^ animals compared to WT and Pyk2^−/−^ mice, though no changes in TBS-soluble p38 MAPK activity (p38 MAPK T180/Y182 normalized to total p38 MAPK) were observed across genotypes (Fig. 10D–F). However, in TBS-insoluble, SDS-soluble fractions that are likely most relevant to aberrant activation and Tau accumulation, the pattern was reversed. While there were no measurable changes in the activity of SDS-soluble LKB1 (pLKB1 S428 normalized to total LKB1) across genotypes, PS19^0/+^;Pyk2^−/−^ animals showed significantly higher activation of SDS-soluble p38 MAPK (p38 MAPK T180/Y182 normalized to total p38 MAPK) compared to WT animals (Fig. 10G–I). When normalized to β-Actin, as a measure of total available active kinase, PS19^0/+^;Pyk2^−/−^ animals displayed increased levels of SDS-soluble p38 MAPK T182/Y182 compared to PS19^0/+^ mice, though we observed no changes in levels of active, SDS-soluble LKB1 (pLKB1 S428 normalized to β-Actin) across genotypes (Fig. 10J–K).

Taken together, these results are consistent with the activities of LKB1 and p38 MAPK contributing to Pyk2’s role in protecting against Tau phosphorylation, Tau pathology, Tau-induced early death, memory impairment and C1q deposition in PS19^0/+^ transgenic animals. While we provide direct evidence for endogenous Pyk2 expression restricting levels of active p38 MAPK, a known Tau kinase, in PS19^0/+^ hippocampus, it remains possible that additional kinases participate in Pyk2-dependent suppression of mutant Tau phenotypes.

## Discussion

The results described here demonstrate a protective role for Pyk2 in suppressing Tau phosphorylation and Tau pathology as well as Tau-induced early death, spatial memory impairment and C1q deposition in PS19^0/+^ transgenic mice. Correlative evidence suggesting a role for Pyk2 in regulating Tau is abundant. Pyk2 (*PTK2B*), which has been repeatedly identified as a genetic LOAD risk factor [3–7], colocalizes with hyperphosphorylated Tau fibrils in AD brains and interacts with Tau when expressed in Hek293 cells [30]. Although at least one group has reported a positive relationship between Pyk2 and Tau phosphorylation in MAPT P301L transgenic mice [31], this association likely depends on Pyk2 over-expression, which in the aforementioned report was driven by the mThy1.2 promoter. Indeed, our results also demonstrate a positive relationship between Pyk2 over-expression and Tau phosphorylation in Hek293T cells, which, as we report here, was dependent on the co-expression of GSK3β.

In order to rule out the potential confound of non-specific kinase-substrate interactions resulting from protein over-expression, we sought to manipulate both the activity and expression of endogenous Pyk2 using iPSC-derived human cortical neurons and PS19^0/+^ (MAPT P301S) transgenic mice. Pharmacological inhibition of basal Pyk2 activity using the selective Pyk2 inhibitor PF-719 increased the phosphorylation of Tau at multiple pathophysiologically-relevant residues in both hippocampal-enriched acute brain slices from PS19^0/+^ mice and in mature iPSC-derived human cortical neurons. As the phosphorylation of these serine and threonine Tau residues occurred independently of any measurable changes to GSK3β activity, other serine/threonine kinases or phosphatases modulated by Pyk2 are likely to be involved.

In support of a protective role for Pyk2 with respect to Tau phosphorylation, genetic deletion of Pyk2 from PS19^0/+^ animals augmented Tau phosphorylation and Tau pathology while exacerbating Tau-induced early death, spatial memory impairment and C1q deposition at synapses. All PS19^0/+^ and PS19^0/+^;Pyk2^−/−^ animals succumbed to a rapid onset of hindlimb paralysis, followed immediately by dramatic reduction in body weight and death. Although the window between onset of gross motor deficits and animal death was short enough to preclude capture by sensorimotor assays, we observed evidence of pronounced Tau pathology extending into the lumbar enlargement of PS19^0/+^ and PS19^0/+^;Pyk2^−/−^ spinal cord, as described previously [48]. The presence of Tau pathology in the spinal cord and the colocalization of AT8-immunofluorescent signal with NeuN-positive motor neurons in the ventral horn, absent measurable sensorimotor impairment, suggests a high threshold for Tau pathological burden in PS19^0/+^ mice before the rapid emergence of hindlimb paralysis.

No memory impairment was observed in PS19^0/+^ animals via MWM at the ages examined, and we were unable to age these mice further without substantial losses in PS19^0/+^;Pyk2^−/−^ animals, whose survivorship was significantly reduced compared PS19^0/+^ mice and likely driven by the spinal cord pathology. Though other groups have observed spatial memory impairment in PS19^0/+^ animals before 9 months, backcrossing these mice to a C57BL/6 J background may have resulted in delayed phenotypic onset. Indeed, phenotypic drift in this transgenic line has been previously reported [74, 75]. We suspect, due to the significant reduction in PSD-95-positive puncta observed in the dentate gyrus, that PS19^0/+^ animals would have likely demonstrated impaired spatial memory if allowed to age further. As no impairments were observed in Pyk2^−/−^ animals, the exacerbations in Tau-induced phenotypes observed in PS19^0/+^;Pyk2^−/−^ animals likely reflect Pyk2’s suppression of Tau phosphorylation per se, resulting in an acceleration of Tau pathology and PS19-associated phenotypes.

Interestingly, impaired spatial memory observed in PS19^0/+^;Pyk2^−/−^ animals occurred independently of any measurable changes in either hippocampal cell layer thickness or synapse density, suggesting that Pyk2 expression in the setting of Tau accumulation modulates synaptic function without affecting synapse number. Observations of impaired synaptic function without associated reductions in synapse density are not without precedence. Synaptic C1q deposition, for example, has previously been found to impair synaptic transmission independent changes in synapse number [61]. Proteomic and biochemical analysis reveal that Pyk2 deletion significantly increased synaptic C1q deposition in PS19^0/+^ hippocampus and cortex, offering a potential mechanistic explanation for impaired memory in PS19^0/+^;Pyk2^−/−^ mice absent any measurable differences in synapse density between PS19^0/+^ and PS19^0/+^;Pyk2^−/−^ animals.

In addition to increased synaptic C1q expression, we observed evidence of substantial disruption to protein translational machinery in PS19^0/+^ animals with Pyk2 genetic deletion. This, combined with the observation that deletion of Pyk2 likely accelerates the emergence of PS19-associated phenotypes including pathology and early death, confounded the use of PS19^0/+^ animals to identify Pyk2-modulated regulators of Tau phosphorylation. To better elucidate the mechanisms by which Pyk2 suppresses Tau phosphorylation, we conducted phospho-proteomic analysis of hippocampal synaptosomes from WT and Pyk2^−/−^ mice and identified 6 putative, proximate regulators of Tau modulated by Pyk2 expression. Of these, we selected LKB1 (STK11) for further validation. Although LKB1 is both necessary and sufficient to induce Tau phosphorylation, LKB1 does not phosphorylate Tau directly [64]. Therefore, in addition to LKB1, we also assessed the ability of Pyk2 to modulate the activity of a known LKB1 substrate and direct kinase of Tau, p38 MAPK [32–38].

Pharmacological inhibition of Pyk2 significantly increased LKB1 and p38 MAPK activity in iPSC-derived human cortical neurons, and the activities of both kinases were significantly elevated in PS19^0/+^;Pyk2^−/−^ hippocampus. Furthermore, Pyk2 deletion significantly increased levels of active p38 MAPK in PS19^0/+^ mice, providing direct evidence that Pyk2 may suppress Tau phosphorylation by limiting hippocampal p38 MAPK activity. Whether Pyk2 acts on these kinases directly or indirectly, and whether any of these interactions can be therapeutically modulated for the treatment of AD has yet to be determined.

Although we investigated LKB1 and p38 MAPK here, it is likely that other kinases also play a role in mediating Pyk2’s suppression of Tau phosphorylation. Like p38 MAPK, MAPK1 has also been identified as a direct Tau kinase, however at least one report shows decreased Tau phosphorylation at S396 with MAPK1 overexpression [76]. Here, we see evidence of a direct relationship between Pyk2 and MAPK1 activity, raising the possibility that Pyk2-mediated activation of MAPK1 may also lead to decreased Tau phosphorylation in PS19^0/+^ animals. MAPKs, a family of kinases that also include JNK, have long been implicated in the pathogenesis of AD [77–80], and a recent large-scale proteomic analysis of postmortem AD brain tissue from the Accelerated Medicines Partnership for Alzheimer’s Disease (AMP-AD) Consortium revealed significant enrichment for proteins related to MAPK signaling and metabolism that strongly correlated with cognitive decline [81].

## Conclusions

The *PTK2B* locus encoding the Pyk2 protein is one of the few validated GWAS risk factors for late-onset Alzheimer’s disease expressed primarily in neurons. Here, we show that in PS19 (MAPT P301S) transgenic mice and in human iPSC-derived neurons, reduced Pyk2 activity exacerbates Tau phosphorylation, pathology and Tau-dependent phenotypes including reduced mouse survival, memory impairment and C1q deposition. Proteomic analysis revealed several proximate regulators of Tau phosphorylation modulated by Pyk2 expression, and direct testing supports a role for LKB1 and, more directly, p38 MAPK in mediating Pyk2’s ability to suppress Tau phosphorylation.

Our previous work has shown that endogenous Pyk2 activation driven by Aβ signaling alters F-actin dynamics and leads to dendritic spine loss with impairment of memory function such that Pyk2-null animals are protected from transgenes driving Aβ pathology [11, 12]. Thus, Pyk2 activity exhibits divergent effects on Aβ versus Tau driven toxicity in AD mouse models; on the one hand contributing to toxic Aβ signaling and, on the other, protecting against Tau phosphorylation and related pathology. The interaction between these divergent Pyk2 effects during the course of AD is not yet delineated, and whether Aβ signaling disrupts Pyk2’s ability to suppress Tau phosphorylation is unknown. As a corollary, any potential therapeutic effect of Pyk2 modulators might depend on disease stage. Pyk2 inhibition, for example, might be most effective during early stages of AD when Aβ-dependent pathophysiology dominates over Tau-dependent pathophysiology.

## Supplementary Information

**Additional file 1.**

## Data Availability

The proteomic datasets analyzed during the current study are available from the corresponding author on reasonable request.

## Abbreviations

Aβ: Amyloid beta
Aβo: Oligomeric amyloid beta
AU: Airy unit
A.U.: Arbitrary units
ACN: Acetonitrile
aCSF: Artificial cerebrospinal fluid
AD: Alzheimer’s disease
ANOVA: Analysis of variance
APP: Amyloid precursor protein
APP/PS1: APPswe/PS1ΔE9
BCA: Bicinchoninic acid
BDNF: Brain-derived neurotrophic factor
BSA: Bovine serum albumin
CD68: Cluster of differentiation 68
CNS: Central nervous system
DAPI: 4′,6-diamidino-2-phenylindole
DEP: Differentially expressed protein
DMEM: Dulbecco’s Modified Eagle’s Medium
DMSO: Dimethyl sulfoxide
DTT: Dithiothreitol
EDTA: Ethylenediaminetetraacetic acid
EN: Enriched
FDR: False discovery rate
FT: Flow through
FTD: Frontotemporal dementia
GFAP: Glial fibrillary acidic protein
GSK3β: Glycogen synthase kinase 3 beta
GWAS: Genome wide association study
Hek293T: Human embryonic kidney 293 with SV40 large T antigen
hiPSC: Human induced pluripotent stem cell
IACUC: Institutional animal care and use committee
IAM: Iodoacetamide
JAX: The Jackson Laboratory
JNK: c-Jun N-terminal kinase
LC-MS/MS: Liquid chromatography with tandem mass spectrometry
LKB1 (STK11): Liver kinase B1 (serine/threonine kinase 11)
LOAD: Late-onset Alzheimer’s disease
LSM: Laser scanning microscope
MAPK1: Mitogen-activated protein kinase 1
MAPT: Microtubule-associated protein tau
MEM: Eagle′s minimum essential medium
mGluR5: Metabotropic glutamate receptor 5
MWM: MORRIS water maze
P2’: Second (crude synaptosomal) pellet
p38 MAPK: p38 mitogen-activated protein kinase
PBS: Phosphate-buffered saline
PFA: Paraformaldehyde
PrP^C^: Cellular prion protein
PS1: Presenilin 1
PSD-95: Post-synaptic density protein 95
PTK2B: Protein tyrosine kinase 2 beta
RIPA: Radioimmunoprecipitation assay buffer
SDS: Sodium dodecyl sulfate
SDS-PAGE: Sodium dodecyl sulfate-polyacrylamide gel electrophoresis
SEM: Standard error of the mean
SMAD: SMA (small worm phenotype) / MAD (mothers against decapentaplegic)
SPE: Solid-phase extraction
TBS: Tris-buffered saline
TBST: Tris-buffered saline with Tween 20
TFA: Trifluoroacetic acid
UPLC: Ultra performance liquid chromatography
WT: Wild type
