# Correction: Alzheimer risk gene product Pyk2 suppresses tau phosphorylation and phenotypic effects of tauopathy

**DOI:** 10.1186/s13024-022-00557-5

**Published:** 2022-08-30

**Authors:** A. Harrison Brody, Sarah Helena Nies, Fulin Guan, Levi M. Smith, Bandhan Mukherjee, Santiago A. Salazar, Suho Lee, Tu Kiet T. Lam, Stephen M. Strittmatter

**Affiliations:** 1 Cellular Neuroscience, Neurodegeneration and Repair Program, Departments of Neurology and Neuroscience, Yale School of Medicine, New Haven, CT USA; 2 Graduate School of Cellular and Molecular Neuroscience, University of Tübingen, D-72074 Tübingen, Germany; 3 Department of Molecular Biophysics and Biochemistry, Yale School of Medicine, New Haven, CT USA; 4 Keck MS & Proteomics Resource, Yale School of Medicine, New Haven, CT USA

**Correction: Mol Neurodegener 17, 32 (2022)**

**https://doi.org/10.1186/s13024-022-00526-y**

The original article [1] contained errors in Figures 3, 4, 7 and 10 which have since been corrected.
