# Supplementary material for: Alzheimer risk gene product Pyk2 suppresses tau phosphorylation and phenotypic effects of tauopathy
Source: Mol Neurodegener. 2022 May 3;17:32. doi: 10.1186/s13024-022-00526-y (PMC9063299; doi:10.1186/s13024-022-00526-y)
Supplement: Supplementary file 1 — Additional file 1. [file 13024_2022_526_MOESM1_ESM.pdf]

ADDITIONAL INFORMATION.

Supplemental Figures 1-7 and Supplemental Table 1.

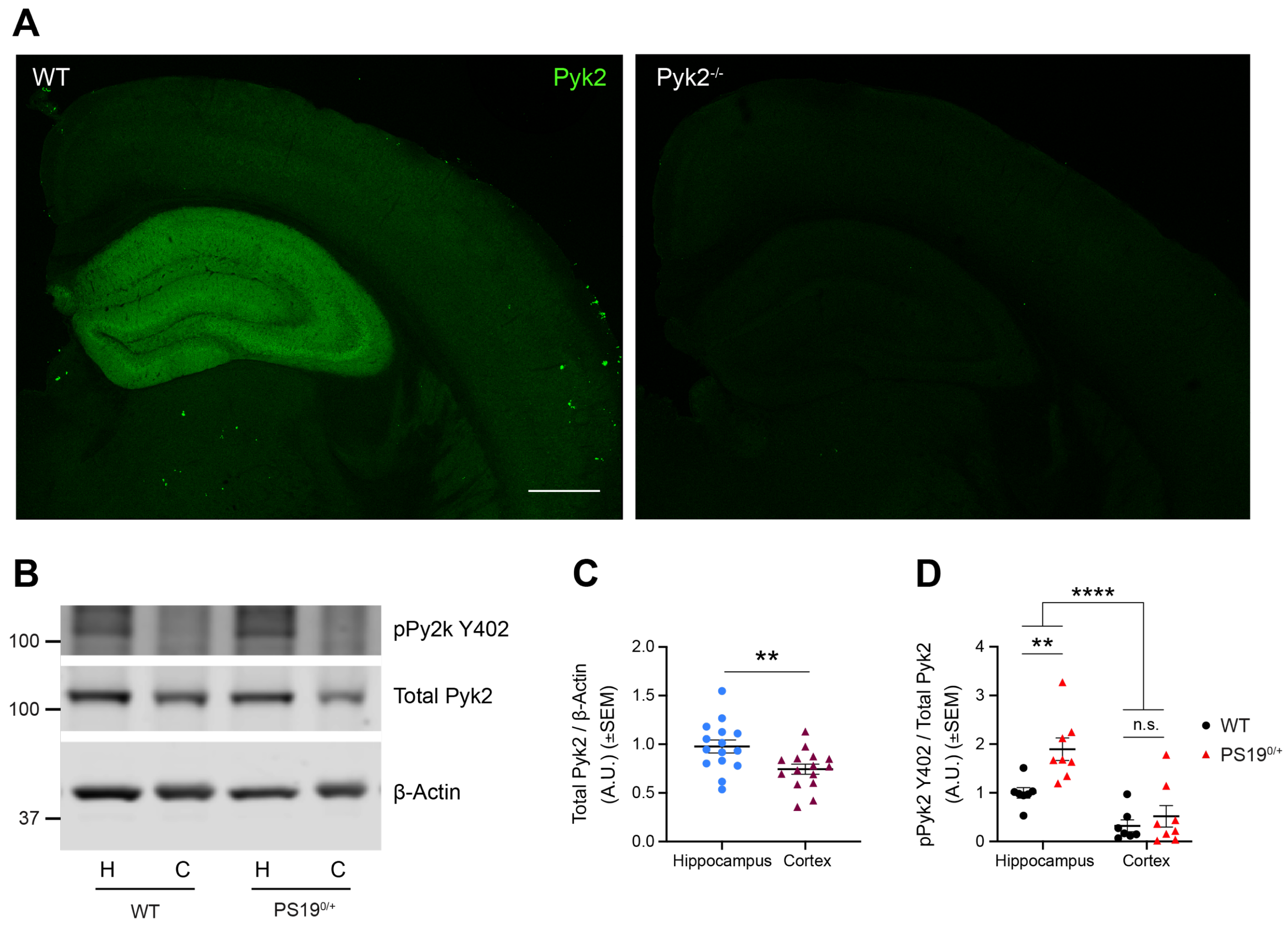

Supplemental Figure 1. Pyk2 expression is predominantly enriched in hippocampus compared to cortex, while PS19-driven Pyk2 activity is confined to hippocampus. **A**, Tiled, immunofluorescent images of Pyk2 immunoreactivity in hippocampus, cortex and thalamus of 9.5–10.5-month-old WT and Pyk2$^{-/-}$ mice. Scale bar, 500 µm. **B**, Representative immunoblot images of TBS-insoluble, SDS-soluble Pyk2 from hippocampus and cortex of 9.5–10.5-month WT and PS19$^{0/+}$ animals. **C** and **D**, Quantification of **B**. **C**, Pyk2 expression is significantly reduced in cortex compared to hippocampus of WT and PS19$^{0/+}$ mice. Data are graphed as mean ± SEM, unpaired two-tailed *t*-test, ***p*<0.01, *n* = 15 mice. **D**, Overall Pyk2 activation (pPyk2 Y402 normalized to total Pyk2) is significantly reduced in cortex compared to hippocampus of WT and PS19$^{0/+}$ mice, while significant tau-induced Pyk2 activation (pPyk2 Y402 normalized to total Pyk2) in PS19$^{0/+}$ compared to WT mice is restricted to hippocampus. Data are graphed as mean ± SEM, two-way ANOVA, ***p*<0.01; unpaired two-tailed t-test, ***p*<0.01, n.s. = not significant (*p* = 0.4657), *n* = 7–8 mice.

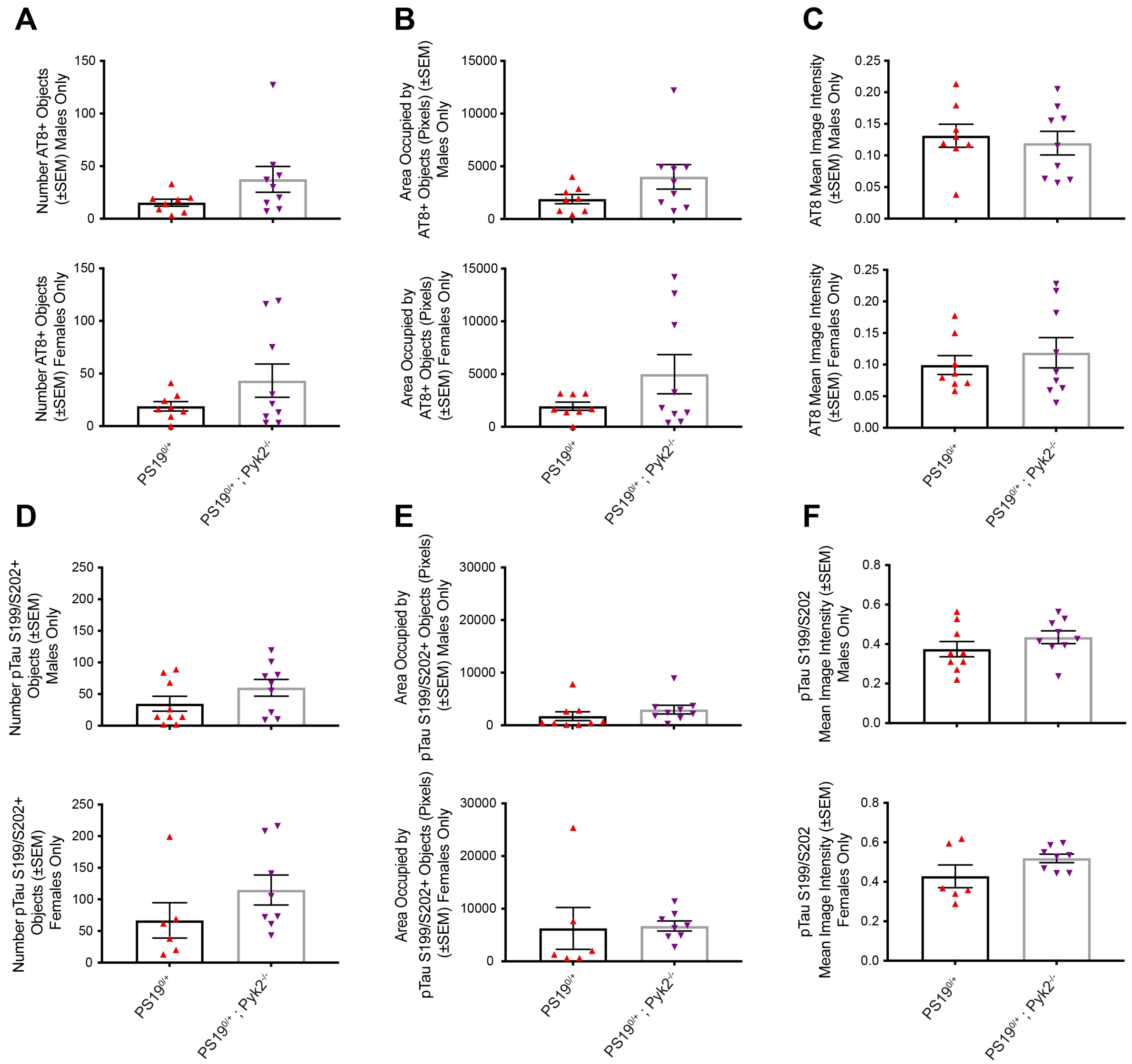

Supplemental Figure 2. Augmented Tau pathology in amygdala of PS19$^{0/+}$;Pyk2$^{-/-}$ compared to PS19$^{0/+}$ mice is driven neither by males nor females alone. **A**–**C**, Quantification of amygdalar AT8 immunoreactivity in 9.5–10.5-month-old PS19$^{0/+}$ and PS19$^{0/+}$;Pyk2$^{-/-}$ animals segregated by sex. No significant differences in the number of AT8-positive cell bodies (objects) (**A**), the area occupied by AT8-postive cell bodies (**B**) or AT8 mean image intensity (**C**) in either male or female PS19$^{0/+}$ and PS19$^{0/+}$;Pyk2$^{-/-}$ animals when segregated by sex. Data are graphed as mean ± SEM, unpaired two-tailed *t*-test, *n* = 8–9 mice. **D**–**F**, Quantification of amygdalar pTau S199/S202 immunoreactivity in 9.5–10.5-month-old PS19$^{0/+}$ and PS19$^{0/+}$;Pyk2$^{-/-}$ animals segregated by sex. No significant differences in number of pTau S199/S202-positive cell bodies (objects) (**D**), area occupied by pTau S199/S202-postive cell bodies (**E**) or pTau S199/S202 mean image intensity (**F**) in male or female PS19$^{0/+}$ and PS19$^{0/+}$;Pyk2$^{-/-}$ animals when segregated by sex. Data are graphed as mean ± SEM, unpaired two-tailed *t*-test, *n* = 6–9 mice.

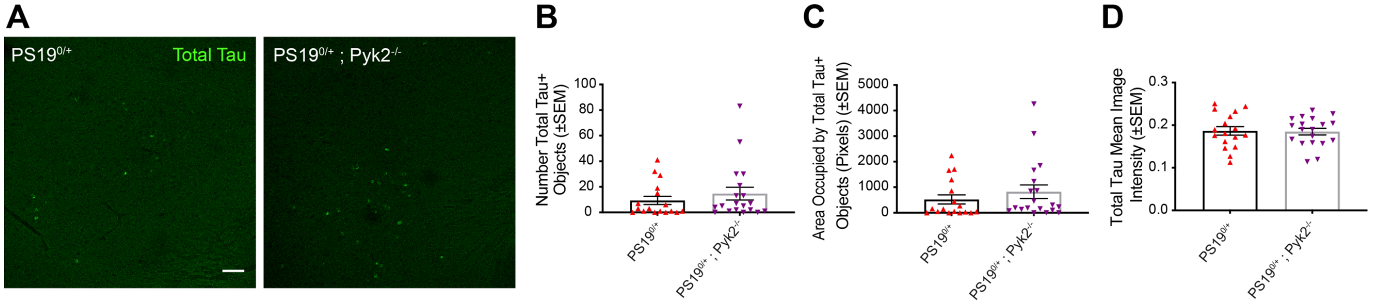

Supplemental Figure 3. Pyk2 deletion fails to result in detectable changes in total Tau immunofluorescence histologically. **A**, Representative immunofluorescent images of total Tau immunoreactivity in amygdala of 9.5–10.5-month-old WT, Pyk2$^{-/-}$, PS19$^{0/+}$ and PS19$^{0/+}$;Pyk2$^{-/-}$ animals. Scale bar, 100 µm. **B**–**D**, Quantification of **A**. Quantification of amygdalar total Tau immunoreactivity reveals no significant changes in the number of total Tau-positive cell bodies (objects) (**B**), the area occupied by those objects (**C**) or in mean image intensity (**D**) between PS19$^{0/+}$ and PS19$^{0/+}$;Pyk2$^{-/-}$ animals. Data are graphed as mean ± SEM, unpaired two-tailed *t*-test, *n* = 17–19 mice.

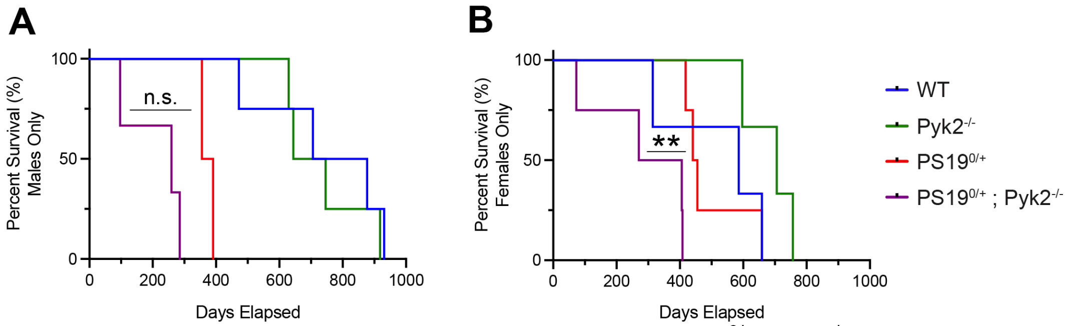

Supplemental Figure 4. Reduced survivorship in PS19$^{0/+}$;Pyk2$^{-/-}$ compared to PS19$^{0/+}$ mice is primarily driven by female animals. **A** and **B**, Kaplan-Meier survival curves of WT, Pyk2$^{-/-}$, PS19$^{0/+}$ and PS19$^{0/+}$;Pyk2$^{-/-}$ animals segregated by sex. Survivorship of male PS19$^{0/+}$;Pyk2$^{-/-}$ mice (median survival, 259 days) is not significantly reduced compared to male PS19$^{0/+}$ animals (median survival, 373 days). Log-rank (Mantel-Cox) test, n.s. = not significant (p = 0.0634), n = 2–3 mice (**A**). Survivorship of female PS19$^{0/+}$;Pyk2$^{-/-}$ mice (median survival, 338 days) is significantly less than that of female PS19$^{0/+}$ animals (median survival, 448 days). Log-rank (Mantel-Cox) test, **p = 0.0067, n = 4 mice (**B**).

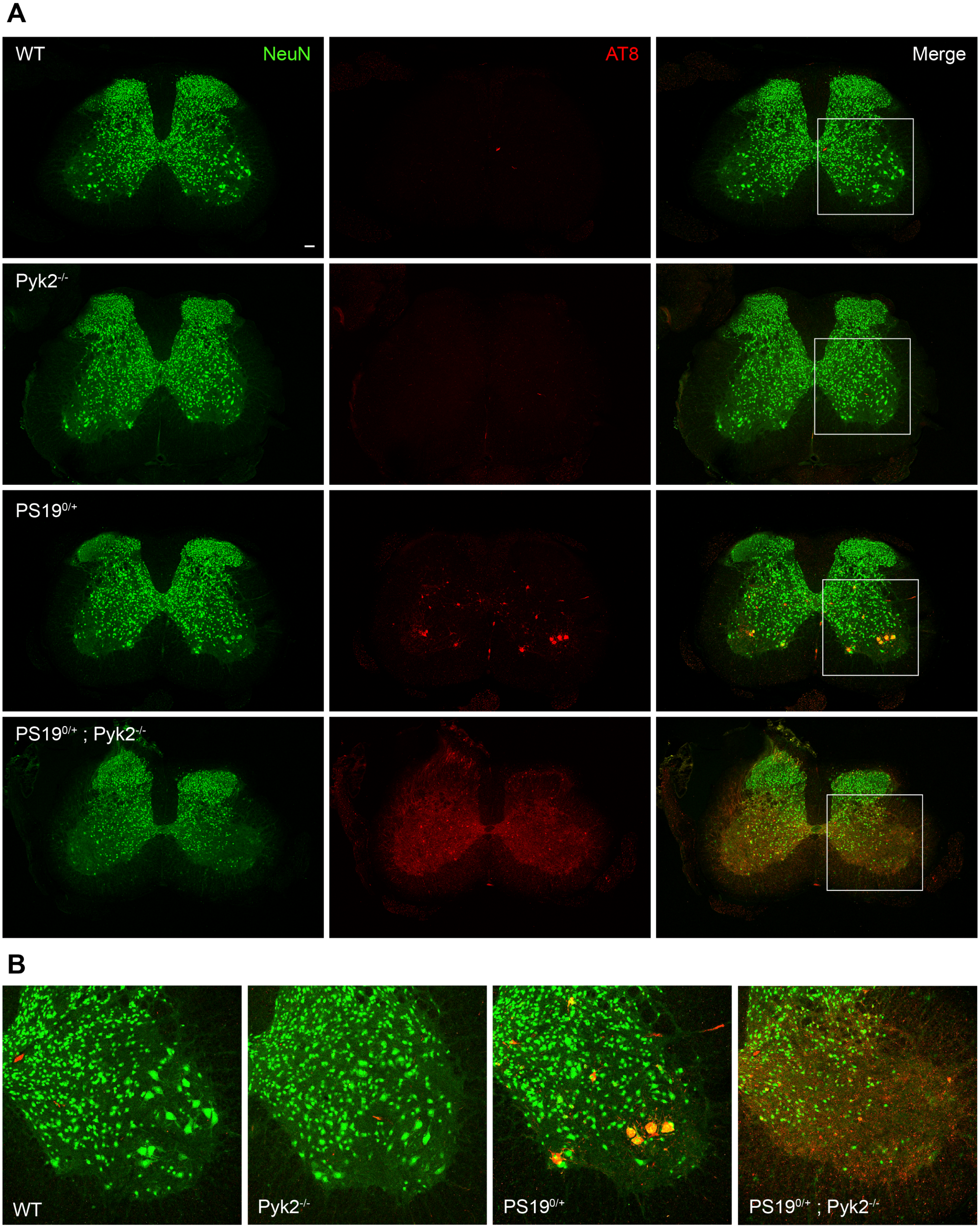

Supplemental Figure 5. PS19-driven Tau pathology is present in spinal cord lumbar enlargement of PS19$^{0/+}$ and PS19$^{0/+}$;Pyk2$^{-/-}$ animals. **A** and **B**, Immunofluorescent images of lumbar spinal cord from 9.5–10.5-month-old WT, Pyk2$^{-/-}$, PS19$^{0/+}$ and PS19$^{0/+}$;Pyk2$^{-/-}$ animals labeled with NeuN (green) and pTau S202/T205 (AT8) (red). Scale bar, 100 µm. **B**, Enlargement of ventral horn shown in **A** (white box)

demonstrating colocalization of NeuN and AT8 immunofluorescence in PS19$^{0/+}$ spinal cord and loss of NeuN-positive neuronal cell bodies in PS19$^{0/+}$;Pyk2$^{-/-}$ spinal cord.

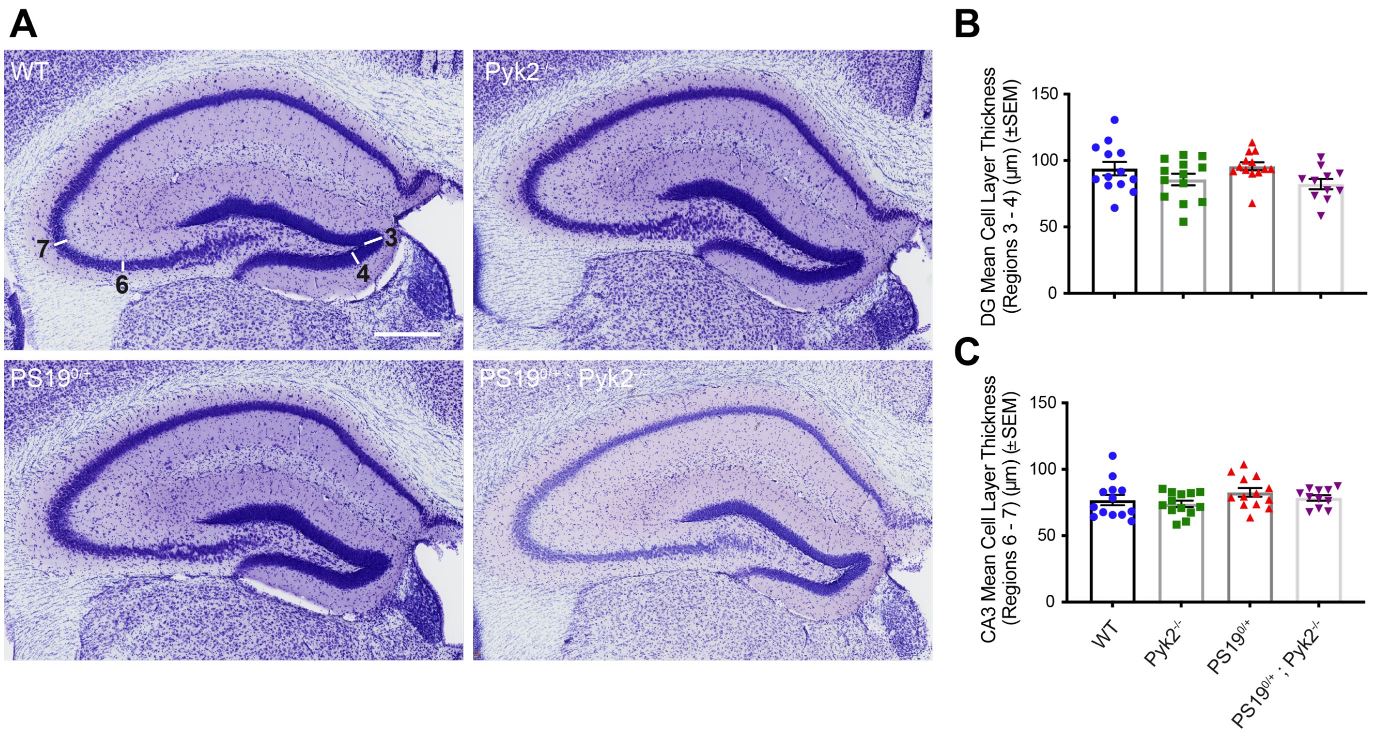

Supplemental Figure 6. No evidence of Tau-induced hippocampal neurodegeneration in PS19$^{0/+}$ or PS19$^{0/+}$;Pyk2$^{-/-}$ animals. **A**, Representative images of cresyl violet-stained sections from 9.5–10.5-month-old WT, Pyk2$^{-/-}$, PS19$^{0/+}$ and PS19$^{0/+}$;Pyk2$^{-/-}$ animals. Scale bar, 400 µm. **B** and **C**, Quantification of hippocampal cell layer thickness in the dentate gyrus (regions 3 and 4) and CA3 (regions 6 and 7) labeled in **A**. There were no significant differences in mean cell layer thickness of the dentate gyrus (**B**) or CA3 (**C**) across genotypes. Data are graphed as mean ± SEM, one-way ANOVA with Tukey's multiple comparisons test, *n* = 11–13 mice.

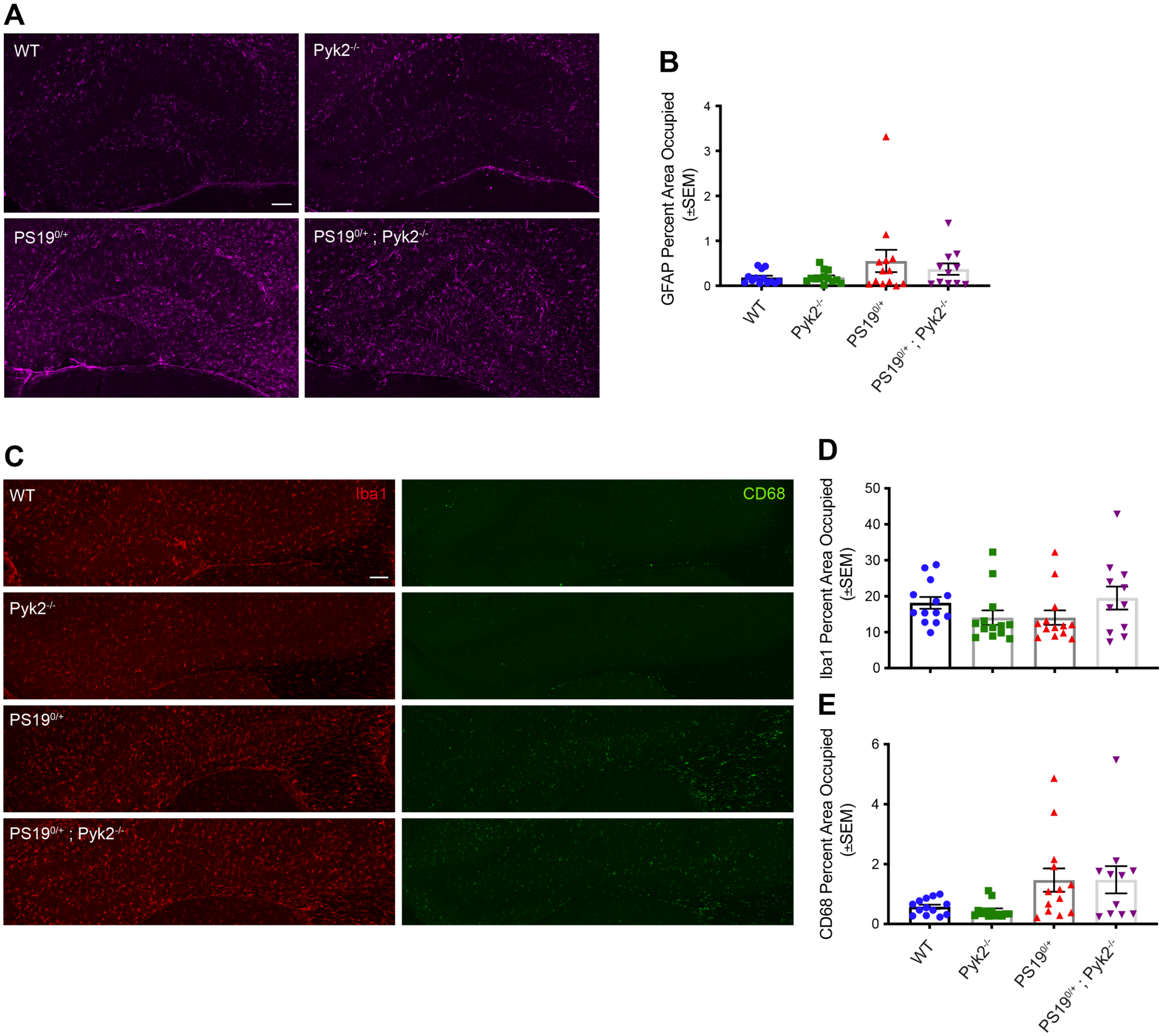

Supplemental Figure 7. Pyk2 deletion fails to result in detectable modulation of Tau-induced gliosis. **A**, Representative tiled, immunofluorescent images of GFAP immunoreactivity in dentate gyrus of 9.5–10.5-month-old WT, Pyk2$^{-/-}$, PS19$^{0/+}$ and PS19$^{0/+}$;Pyk2$^{-/-}$ animals. Scale bar, 100 μm. **B**, Quantification of **A**. There were no significant differences in astrogliosis (GFAP percent area occupied) across genotypes (**B**). Data are graphed as mean ± SEM, one-way ANOVA with Tukey's multiple comparisons test, *n* = 11–13 mice. **C**, Representative tiled, immunofluorescent images of Iba1 (red) and CD68 (green) immunoreactivity in hippocampus of 9.5–10.5-month-old WT, Pyk2$^{-/-}$, PS19$^{0/+}$ and PS19$^{0/+}$;Pyk2$^{-/-}$ animals. Scale bar, 100 μm. **D** and **E**, Quantification of **C**. There were no significant differences in total hippocampal microglia (Iba1 percent area occupied) (**D**) nor activated hippocampal microglia (CD68 percent area occupied) (**E**) across genotypes. Data are graphed as mean ± SEM, one-way ANOVA with Tukey's multiple comparisons test, *n* = 11–13 mice.

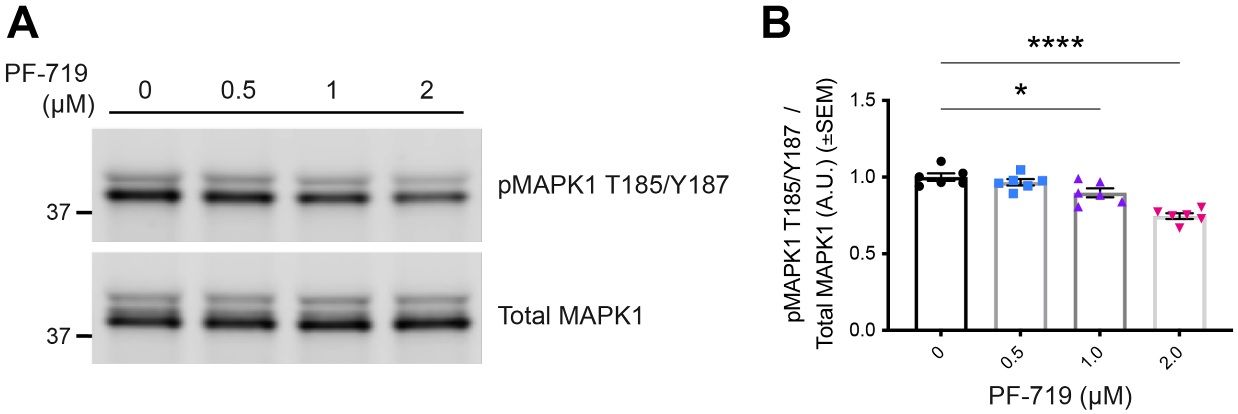

Supplemental Figure 8. Pyk2 activates MAPK1. **A** and **B**, Pyk2 was pharmacologically inhibited in iPSC-derived human cortical neurons (90–100 days post terminal differentiation) (same as shown in Figure 2e–i) using PF-719 at the concentrations indicated. **A**, Representative immunoblot images of lysates from PF-719-treated iPSC-derived human cortical neurons. **B**, Quantification of **A**. Pyk2 inhibition significantly decreased MAPK1 activity (pMAPK1 T185/Y187 normalized to total MAPK1) at 1.0 and 2.0 μM PF-719 (**B**). Data are graphed as mean ± SEM, one-way ANOVA with Dunnett's multiple comparisons test, *p<0.05, ****p<0.0001, n = 6.

Supplemental Table 1. Kyoto Encyclopedia of Genes and Genomes (KEGG) enrichment for total protein hits across proteomic analyses. Only one node, “pathways of neurodegeneration,” was identified from hits generated from the $PS19^{0/+}$ vs $PS19^{0/+}$;$Pyk2^{-/-}$ analysis, while no pathways were identified for the WT vs $Pyk2^{-/-}$ analysis.

Fraction	Analysis	% Associated Genes	Associated Genes Found	Number of Genes	Pathway Term	KEGG ID	Term P-Value	Term P-Value Corrected with Bonferroni Step Down
Total Protein	WT vs $Pyk2^{-/-}$	NA	NA	NA	NA	NA	NA	NA
	$PS19^{0/+}$ vs $PS19^{0/+}$;$Pyk2^{-/-}$	1.89	CSNK2B, CYTB, DLG4, FUS, MTOR, PLCB1, PLCB3, RYR1, UQCR10	9	Pathways of neurodegeneration	KEGG:05022	0.187479957	0.187479957
